# Ulcerative colitis, but not Crohn’s disease, displays demographically and clinically driven mucosal immune heterogeneity: implications for patient stratification

**DOI:** 10.3389/fimmu.2026.1965046

**Published:** 2026-09-24

**Authors:** Ángel De Prado, Irene Soleto, Carolina G. de Castro, Montse Baldán-Martín, Alejandro G. del Hierro, Paula J. Martínez, Elisa Arribas-Rodríguez, Fabio Suarez-Trujillo, Aida Fiz-López, Álvaro Martín-Muñoz, Alfredo J. Lucendo, Cristina Rodríguez Gutiérrez, Ana Gutiérrez-Casbas, Eduardo Martín Arranz, Luis Bujanda, Iago Rodríguez-Lago, Ruth M. de Francisco García, Eva Iglesias-Flores, Fernando Bermejo, Manuel Van Domselaar, María José García, Luis Fernández-Salazar, Francisco J. García-Alonso, Xavier Calvet Calvo, Leyanira Torrealba, Iria Bastón-Rey, Héctor Pallares Manrique, Yolanda Ber Nieto, Eduardo Leo Carnerero, María Chaparro, Javier P. Gisbert, David Bernardo

**Affiliations:** 1 Mucosal Immunology Lab, Institute of Biomedicine and Molecular Genetics from Valladolid (IBGM Universidad de Valladolid-CSIC), Valladolid, Spain; 2 Servicio de Aparato Digestivo, Hospital Universitario de La Princesa, Instituto de Investigación Sanitaria Princesa (IIS-Princesa), Centro de Investigación Biomédica en Red de Enfermedades Hepáticas y Digestivas (CIBEREHD), Madrid, Spain; 3 Flow Cytometry Unit, Institute of Biomedicine and Molecular Genetics (IBGM), Universidad de Valladolid-CSIC, Valladolid, Spain; 4 Hospital General de Tomelloso, Instituto de Investigación Sanitaria de Castilla-La Mancha (IDISCAM), CIBEREHD, Dept. de Gastroenterology, Ciudad Real, Spain; 5 Hospital Universitario de Navarra, Instituto de Investigación de Navarra IdiSNAP, Pamplona, Spain; 6 Hospital Universitario Dr Balmis de Alicante, Spain (Gastroenterology Department), ISABIAL, CIBEREHD, Alicante, Spain; 7 Hospital Universitario La Paz, Instituto de Investigación Sanitaria La Paz (IdiPaz), Gastroenterology Unit, Madrid, Spain; 8 Department of Hepatology and Gastroenterology, Biogipuzkoa Health Research Institute - Donostia University Hospital, Universidad del País Vasco (EHU), Centro de Investigación Biomédica en Red de Enfermedades Hepáticas y Digestivas (CIBERehd), San Sebastián, Spain; 9 Hospital Universitario de Galdakao, Biocruces Bizkaia HRI, Gastroenterology, Vizcaya, Spain; 10 Hospital Universitario Central de Asturias, Instituto de Investigación Sanitaria del Principado de Asturias (ISPA), Oviedo, Spain; 11 Servicio de Aparato digestivo, Hospital Universitario Reina Sofía, IMIBIC, UCO, Centro de Investigación Biomédica en Red de Enfermedades Hepáticas y Digestivas (CIBEREHD), Córdoba, Spain; 12 Hospital Universitario de Fuenlabrada, Gastroenterology, Madrid, Spain; 13 Hospital Universitario de Torrejón, Madrid, Spain; 14 Hospital Universitario Marqués de Valdecilla and IDIVAL, Gastroenterology, Santander, Spain; 15 Hospital Clínico Universitario de Valladolid, Universidad de Valladolid, Valladolid, Spain; 16 Instituto de Investigación Biosanitaria de Valladolid (IBioVALL), Valladolid, Spain; 17 Hospital Universitario Rio Hortega, Valladolid, Spain; 18 Gastroenterology Department, Parc Taulí Hospital Universitari, Institut d’Investigació i Innovació, Parc Taulí (I3PT-CERCA), Sabadell, Catalunya, Departament de Medicina, Universitat Autònoma de Barcelona, CIBERehd, Centro de Investigación Biomédica en Red de Enfermedades Hepáticas y Digestivas (CIBEREHD), Instituto de Salud Carlos III, Madrid, Spain; 19 Hospital Universitario Dr. Josep Trueta, Girona, Spain; 20 Hospital Clínico Universitario de Santiago, Gastroenterology and Hepatology, Santiago de Compostela, Spain; 21 Hospital Juan Ramón Jiménez, Huelva, Spain; 22 Hospital San Jorge, Huesca, Spain; 23 Hospital Universitario Virgen del Rocío, Sevilla, Spain; 24 Centro de Investigaciones Biomédicas en Red de Enfermedades Infecciosas (CIBERINFEC), Madrid, Spain

**Keywords:** Crohn‘ s disease, immunophenotyping, inflammatory bowel disease, spectral cytometry, ulcerative colitis

## Abstract

**Background:**

Inflammatory bowel disease (IBD) encompasses Crohn’s disease (CD) and ulcerative colitis (UC), two conditions with distinct immunopathological profiles despite shared inflammatory pathways. Comparative mucosal and systemic immune characterisation across disease subtypes and clinical variables remains limited. This study aimed to map leukocyte dysregulation in tissue and blood from patients with CD and UC compared to controls.

**Methods:**

Intestinal tissue and blood samples were obtained from 35 controls and endoscopically active IBD patients, including 54 with CD and 74 with UC. Leukocyte subsets were identified using a 29-marker spectral cytometry panel combining hierarchical gating with unsupervised clustering and dimensionality reduction. Immune composition was stratified by disease location, biological sex, age and endoscopic severity.

**Results:**

Hierarchical gating identified 41 mucosal and 42 circulating immune subsets; unsupervised clustering resolved 51 mucosal and 62 blood clusters. Both diseases displayed dysregulated mucosal immunity relative to controls but with distinct signatures: while CD was mainly T-cell biased, UC showed a mixed infiltrate with an increased relative proportion of NK, NKT and IgG^+^ B-cells. UC also exhibited substantially greater immunological heterogeneity than CD when stratified by biological sex, age and severity, with effects predominantly confined to the mucosa. Ileal and colonic CD showed minor immunological differences.

**Conclusions:**

UC displayed more prominent subgroup-associated mucosal immune heterogeneity than CD, particularly according to biological sex, age and endoscopic severity. These findings support tissue-resolved patient stratification in UC and highlight the limited sensitivity of blood-based biomarkers to reflect mucosal immune alterations.

## Introduction

Inflammatory bowel disease (IBD) comprises chronic, relapsing-remitting inflammatory conditions of the gastrointestinal tract, with rising incidence and prevalence worldwide, particularly in industrialised regions (1–3).

IBD presents with diarrhoea, abdominal pain, bleeding, weight loss, and extra-intestinal manifestations, with variable course, severity and therapeutic response (4, 5). Its two main forms, Crohn’s disease (CD) and ulcerative colitis (UC), differ clinically and pathologically: CD may affect any gastrointestinal segment and is typically transmural, whereas UC is limited to continuous mucosal inflammation of the colon (6, 7).

IBD aetiology is multifactorial, involving genetic predisposition, environmental triggers, alterations of the gut microbiota, and a dysregulated immune response (8–10). Aberrant innate and adaptive immune activation is central to pathogenesis (11, 12), yet how immune cell subsets differ between CD and UC remains incompletely defined.

We have therefore performed high-dimensional spectral and computational cytometry of mucosal and circulating immune cells from endoscopically inflamed CD and UC patients, stratified by disease location, severity, biological sex and age. This approach identifies disease-specific immune signatures and exploratory subgroup-associated immune patterns with potential relevance for future biomarker discovery and patient stratification.

## Materials and methods

### Patient demographics

Biological samples were obtained from a total of 35 controls [17 males, 18 females, with a median age of 57 years (interquartile range 31–75 years)], 54 patients with CD [33 males, 21 females, with a median age of 48 years (interquartile range 22–74 years)], and 74 patients with UC ([41 males, 33 females, with a median age of 50 years (interquartile range 21–80 years)].

Control samples were obtained at Hospital Universitario de La Princesa (Madrid, Spain) during colonoscopy for colorectal cancer screening, anaemia, or abdominal pain evaluation. Control subjects had no endoscopic or histological inflammation and no known malignant, autoimmune or inflammatory disease. IBD patients were recruited within the framework of the BioIBD protocol (Ethics Committee Code: GIS-2018-BioIBD, Hospital Universitario de La Princesa), which reflects a cohort of real-world population of endoscopically active IBD patients sampled before the first administration of a biologic therapy with a specific mechanism of action. The study protocol was registered in both European and United States clinical trial registries (EudraCT number 2018-002925–47 and ClinicalTrials.gov identifier NCT03885713). Colonoscopies were performed in all cases as part of the study protocol, and written informed consent was obtained from all participants prior to inclusion. All IBD patients showed endoscopic evidence of active inflammation at the time of sample collection.

In patients with CD, endoscopic disease activity was assessed using the Simplified Endoscopic Activity Score for Crohn’s Disease (SES-CD) (13–15), defining active disease as SES-CD ≥ 3. In patients with UC, disease activity was evaluated using the endoscopic subscore of the Mayo Clinic Index (16, 17), with an endoscopic subscore ≥ 2 considered indicative of active disease. Further information from IBD patients regarding sociodemographic variables (including age, biological sex, and disease location), smoking habit and disease severity are summarised in **Table 1**.

**Table 1:** Demographic and clinical characteristics of the cohorts.

Characteristics	Crohn’s Disease (CD) (n=54)	Ulcerative colitis (UC) (n=74)	P-value
**Female sex, n (%)**	21 (38.9)	33 (44.6)	0.550
**Non-smokers, n (%)**	44 (81.5)	70 (94.6)	0.027
**Age, years, mean (range)**	47.8 (22-74)	50.1 (21-80)	0.610
Location			
Ileal	36		
Colonic	18		
CD endoscopic severitySES-CD)			
Mild 3-6	17		
Moderate 7-15	30		
Severe ≥16	7		
UC endoscopic severity (Mayo score)			
Moderate (Mayo 2)		28	
Severe (Mayo 3)		46	

Values are presented as n (%) or mean (range). P-values correspond to comparisons between Crohn’s disease and ulcerative colitis; SES-CD, Simple Endoscopic Score for Crohn’s Disease.

Bold values indicate statistical significance (p < 0.05).

### Biological samples

Blood and intestinal mucosal samples were obtained from each individual. Peripheral blood mononuclear cells (PBMCs) were isolated from 9 mL lithium heparin blood by Ficoll-Paque PLUS (Amersham) centrifugation and cryopreserved in 90% foetal bovine serum, 10% sterile dimethyl sulfoxide. 8 biopsies were obtained per individual: left/distal colon in controls, affected colon in UC and colonic CD, and terminal ileum in ileal CD. Biopsies were preserved in ice-chilled complete medium and cryopreserved as previously reported (18).

### Sample processing

Thawed, mucosal biopsies were processed to obtain lamina propria mononuclear cells (LPMCs) using protocols optimised by our group (19–21). Briefly, mucus, bacteria and epithelial layers were removed by two 30-minute Hanks’ balanced salt solution (HBSS) incubations containing 1 mM dithiothreitol (DTT) and 1 mM ethylenediaminetetraacetic acid (EDTA). Tissue was then digested by three 30-minute incubations with 1 mg/mL Collagenase D, 20 μg/mL Liberase and 25 U/mL Benzonase, filtered through a 100-μm strainer and centrifuged to recover LPMCs.

In contrast to LPMCs, PBMCs were thawed, washed, and prepared for staining directly without additional processing steps.

### Antibody staining and cell acquisition

LPMCs and PBMCs were stained with a panel of 29 monoclonal antibodies (**Supplementary Table 1**) based on the OMIP-069 panel (22). Dead cells were excluded using Live/Dead fixable blue staining, followed by Brilliant Stain Buffer and True-Stain Monocyte Blocker. IgG and CXCR5 were stained first, followed by the remaining antibodies. Cells were washed and fixed with 1% paraformaldehyde and acquired within 24 hours on a 5-laser, 67-detector Aurora spectral cytometer (Cytek). Instrument performance was monitored according to the manufacturer’s quality-control procedures before acquisition. Manual gating followed a predefined strategy based on fluorescence minus one (FMO) controls and was applied uniformly across samples.

### Hierarchical cytometry analysis

PBMCs and LPMCs were analysed by hierarchical gating within singlet viable CD45^+^ cells (**Supplementary Figures 1A, B**). Major immune cell populations of the immune system in both compartments, like basophils, antigen-presenting cells (APC), including monocytes, macrophages and conventional dendritic cells (cDC), as well as natural killer (NK) cells, innate lymphoid cells, B-cells, and T-cells, were identified, yielding 41 tissue and 42 blood immune subsets. In addition, several subsets were identified within each population. Gates were established using FMO controls Major immune-cell lineages were expressed as relative frequencies within total singlet viable CD45^+^ leukocytes, whereas downstream phenotypic subsets were expressed relative to their corresponding parental populations. Therefore, the reported changes reflect relative frequencies or proportions rather than absolute cell numbers.

### Computational or non-supervised cytometry analysis

Data were analysed within each tissue (blood or intestinal mucosa) based on the patient cohort from which they were derived (controls, UC and CD). In addition, cells within each tissue (LPMCs or PBMCs) were further analysed, within each cohort, based on age, biological sex, and disease severity as shown in **Supplementary Tables 2** and **3**, respectively, which also provide their sample sizes.

In all cases, flow cytometry files (.fcs) were analysed using the OMIQ Data Science platform-recommended (Omiq, Inc. 2022) scales, parameters and cofactors, following our previously described workflow (21, 23).

Briefly, the data-cleaning PeacoQC algorithm was applied to remove outlier events related to abnormal flow behaviour, including clogs and other acquisition instabilities. Subsequently, a manual discard was performed to eliminate cell debris and doublets and to select viable leucocytes (CD45^+^ cells), which were used for subsequent analysis. Cohort representation was balanced during sampling before UMAP visualisation.

UMAP was applied for exploratory analysis after random subsampling to 5,000,000 cells, ensuring equal cohort representation. This method embeds high-dimensional marker-expression data into two dimensions, grouping cells with similar phenotypes.

FlowSOM was then used for unsupervised clustering, aggregating cells into metaclusters based on marker-expression profiles and enabling identification of expected and unexpected populations.

Metaclusters were further subdivided into clusters representing phenotypic and functional immune subsets in blood and tissue. Clustered heatmaps summarised marker expression. Clusters with less than 1,000 events or with more than 50% of the events from a single patient were excluded.

### Statistical analysis

For the computational cytometry data, differential cluster abundance was assessed using edgeR algorithm. Cluster counts were compared between groups, and volcano plots were used to represent log_2_ fold change and FDR-adjusted significance. Comparisons were performed between predefined groups, including controls, CD and UC, as well as stratified analyses according to disease location, biological sex, age and endoscopic severity. Using a modified gating strategy for the OMIP-69 panel, the percentages within the total viable leukocyte fraction (CD45^+^) of the selected clusters identified in the previous computational analysis were obtained and further analysed using GraphPad Prism 9 software (San Diego, CA, USA).

Quantitative variables were expressed as the mean ± standard deviation (SD). For comparisons between groups, parametric tests were applied when data followed a normal distribution. A significance level of p < 0.05 was established for all analyses, with False Discovery Rate (FDR) values below this threshold considered statistically significant. Volcano plots report log_2_ fold change and FDR-adjusted significance for differential cluster abundance between groups.

## Results

### A hierarchical gating analysis reveals an abnormal mucosal and circulating immune fingerprint in both CD and UC

Within the human intestinal mucosa, a total of 41 immune cells subsets were identified using classical gating approaches within total LPMCs (as shown in **Supplementary Figure 1A**), showing in **Figure 1A** a summary of their relative proportion. Briefly, UC patients displayed higher frequencies of CD8^+^ T-cells (typically of an effector memory phenotype) which was not mirrored by CD patients, while both IBD cohorts displayed a trend towards higher frequencies of NK and natural killer T (NKT) cells. In addition, UC patients also carried a higher proportion of mucosal B-cells while CD was more characterised by higher frequencies of type 2 cDC and a lower proportion of macrophages.

**Figure 1:**
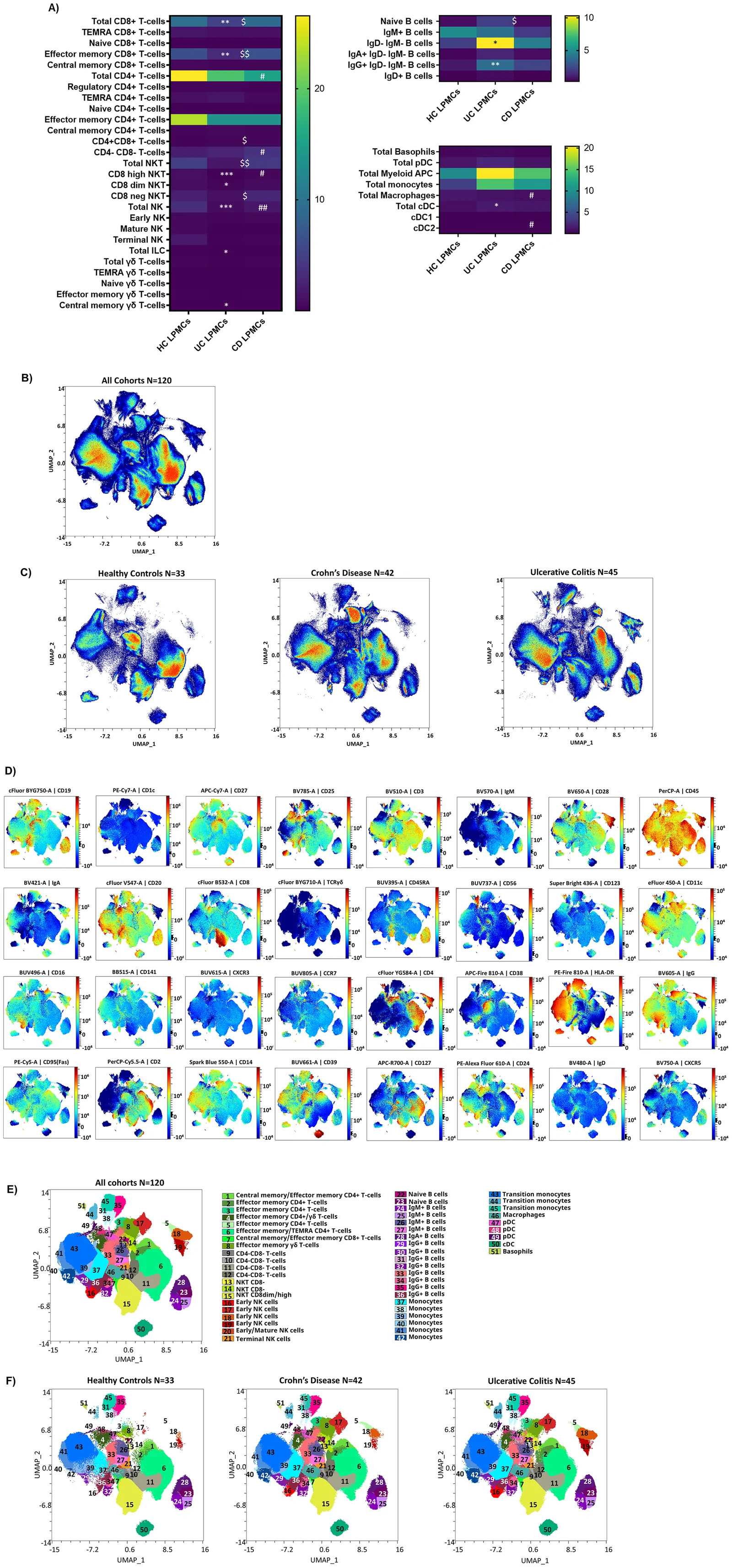
The human intestinal immunome in inflammatory bowel disease. **(A)** The relative proportion from a total of 41 different immune cell subsets (identified by classical gating strategies as shown in **Supplementary Figure 1**) within total lamina propria mononuclear cells (LPMCs) from healthy controls (HC), patients with endoscopically active ulcerative colitis (UC) and Crohn´s disease (CD), referred to the total number of mucosal singlet viable CD45^+^ cells, is shown. **(B)** A UMAP representation using all events within singlet viable CD45^+^ cells was also determined using all the patients, showing in **(C)** the individual contribution of each cohort. The relative expression of each marker within the UMAP is also shown in **(D)** while panel **(E)** displays the location of each of the 51 different mucosal clusters identified following FlowSOM analysis as further detailed in **Supplementary Table 4**. Last the density of each cluster within each cohort is displayed in panel **(F)** One-Way ANOVA was applied in Panel A in order to display the differences among the 3 studied cohorts following subsequent *post hoc* correction (Tukey correction). P-value < 0.05 was considered as statistically significant. Symbols refer to the comparison among cohorts, using * to compare UC and HC, ^$^ to compare CD and HC, and ^#^ to compare UC and CD. One symbol means p < 0.05; two symbols p < 0.01; and three symbols p < 0.001.

Given nevertheless that the use of complex cytometry panels can provide deeper insights into the characterization of a given sample by means of non-hierarchical gating approaches (24) a subsequent UMAP analysis was also performed (**Figure 1B**), revealing the presence of immune differences among the 3 studied cohorts (**Figure 1C**), showing in **Figure 1D** the relative proportion of each marker throughout the UMAP. That allowed, following the FlowSOM analysis, the identification of a total of 51 different mucosal clusters as further detailed in **Supplementary Table 4**, and which are summarised in **Figure 1E** hence providing a visual representation of their relative abundance in the mucosa as in **Figure 1F**.

The same approach was also performed in the circulating precursors from the same patients, where we could identify a total of 42 different subsets. Of note, and opposed to the mucosa where IBD patients displayed a none overlapping immune profile with differences on their relative proportion of different T-cell subsets and naïve B-cells between CD and UC patients (**Figure 1A**), the circulating immune profile was nevertheless quite consistent among both CD and UC patients compared to controls (**Figure 2A**), being characterised by an increased relative frequency of T-cell subsets (mainly of a helper phenotype) and a reduced relative proportion of B cell and APC subsets. As above, a UMAP analysis was also performed (**Figures 2B–D**) which allowed the visualization of a total of 62 different clusters (further described in **Supplementary Table 5**) in **Figures 2E, F**.

**Figure 2:**
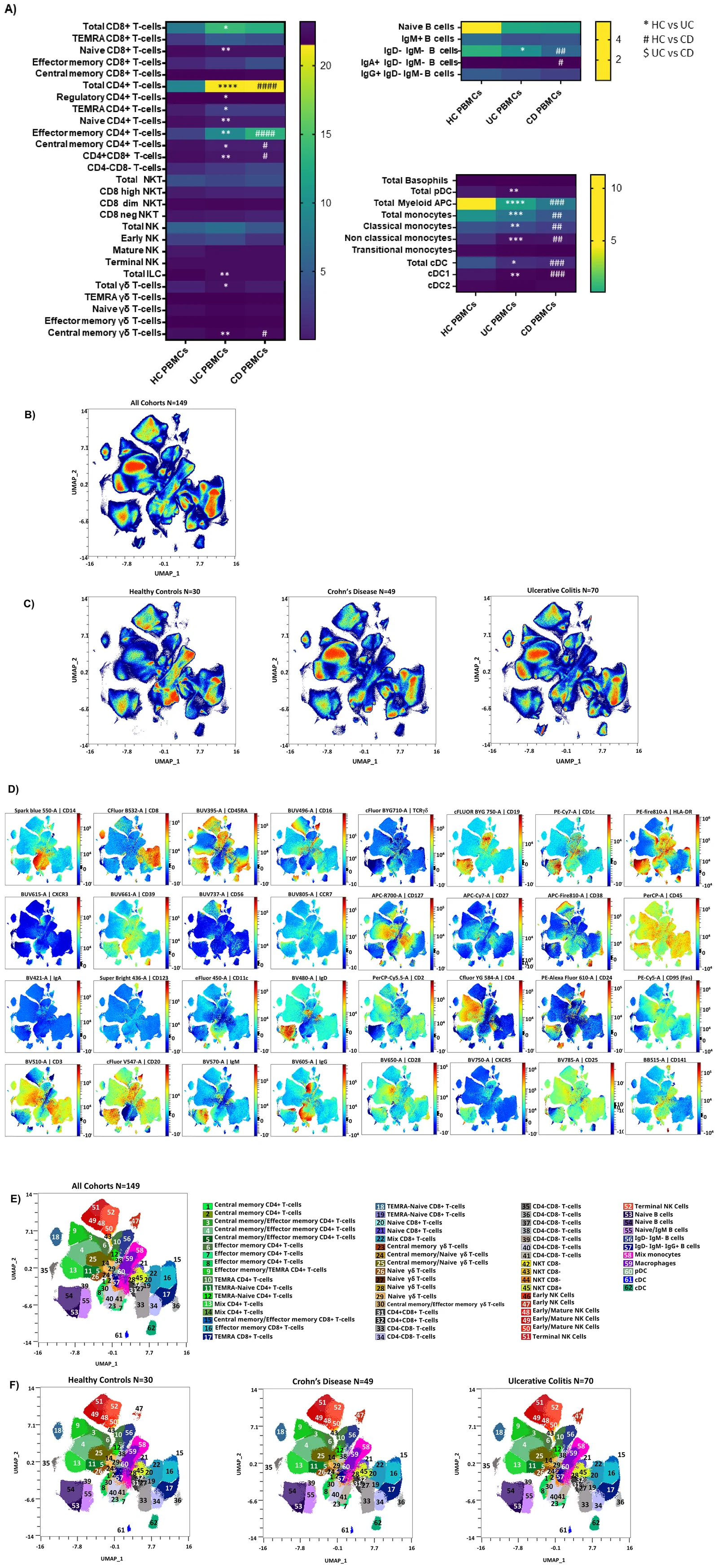
The circulating immune system in inflammatory bowel disease. **(A)** The relative proportion from a total of 42 different immune cell subsets (identified by classical gating strategies as shown in **Supplementary Figure 1**) within total peripheral blood mononuclear cells (PBMCs) from healthy controls (HC), patients with endoscopically active ulcerative colitis (UC) and Crohn´s disease (CD), referred to the total number of mucosal singlet viable CD45^+^ cells, is shown. **(B)** A UMAP representation using all events within singlet viable CD45^+^ cells was also determined using all the patients, showing in **(C)** the individual contribution of each cohort. The relative expression of each marker withing the UMAP is also shown in **(D)**, while panel **(E)** displays the location of each of the 62 different blood clusters identified following FlowSOM analysis as further detailed in **Supplementary Table 5**. Last the density of each cluster within each cohort is displayed in panel **(F)**. One-Way ANOVA was applied in Panel **(A)** in order to display the differences among the 3 studied cohorts following subsequent *ad-hoc* correction (Tukey correction). P-value <0.05 was considered as statistically significant. Symbols refer to the comparison among cohorts, using * to compare UC and HC, ^$^ to compare CD and HC, and ^#^ to compare UC and CD. One symbol means p < 0.05; two symbols p < 0.01; three symbols p < 0.001; and four symbols p < 0.0001.

### CD patients displayed a T-cell biased immune profile whereas UC showed a mixed mucosal immune profile

Having described both the mucosal and the circulating immune system in IBD patients, we next performed a comprehensive characterisation of the mucosal immune fingerprint from both CD and UC patients. Moreover, and given that CD, as opposed to UC, can affect any area of the human gut, and since the properties of the immune system systematically change through its length (25, 26), we first analysed colonic CD referred to colonic controls. A volcano plot analysis (**Figure 3A**) revealed a markedly altered immune response in patients with colonic CD characterised by a markedly increased proportion of helper T-cells of a memory phenotype (excluding TEMRA cells), together with non-classical (CD4^-^CD8^-^) T-cells, cells of a cytotoxic phenotype (including NK and NKT cells), as well as IgG^+^ B-cells (which translated into a specific depletion of non IgG^+^ B-cells). **Figures 3B, C** provide a more visual representation of these differences. Indeed, and in agreement with these observations, the analysis of the circulating precursors from these patients revealed a reduced relative proportion of circulating helper T-cells of a memory phenotype (**Figures 3D–F**) consistent with their higher relative abundance in the colonic mucosa.

**Figure 3:**
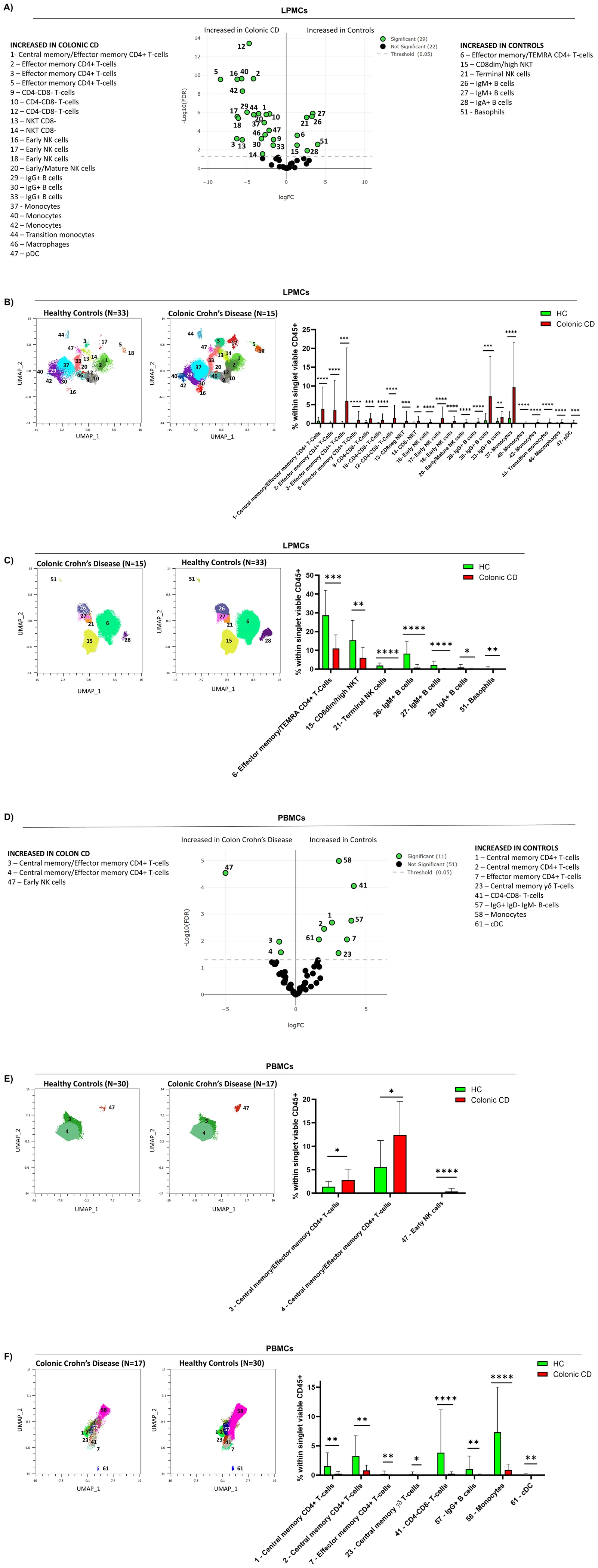
The mucosal and circulating immunome in patients with colonic Crohn´s disease. **(A)** Volcano plot showing differential cluster abundance between patients colonic Crohn’s disease (CD) and controls within lamina propria mononuclear cells (LPMCs). Each point represents a FlowSOM-defined cluster; the x-axis indicates log_2_ fold change between groups, and the y-axis shows statistical significance (–log_10_ FDR). Clusters reaching statistical significance after multiple-testing correction are highlighted. **(B)** Clusters significantly enriched in colonic CD are projected onto the global UMAP representation, allowing visualization of their spatial distribution within the overall cellular landscape. Bar plots display the relative abundance of these clusters in each group. **(C)** Clusters significantly enriched in healthy controls are projected onto the global UMAP, together with bar plots showing their relative abundance across groups. The same volcano approach was performed in the circulating clusters obtained within peripheral blood mononuclear cells (PBMCs) as shown in **(D, E)** Circulating clusters significantly enriched in colonic CD are projected onto the global UMAP representation, allowing visualization of their spatial distribution within the overall PBMCs landscape. Bar plots display the relative abundance of these clusters in each group. **(F)** Clusters significantly enriched in healthy controls are projected onto the global UMAP, together with bar plots showing their relative abundance across groups. Differential abundance analysis was performed using edgeR, applying a generalised linear model with multiple-testing correction by the Benjamini–Hochberg FDR method in panels **(A, D)**. Unpaired two-tailed t-test was applied in panels **(B, C, E, F)**. P-value < 0.05 was considered as statistically significant (*p < 0.05; **p < 0.01; ***p < 0.001; ****p < 0.0001).

As for the patients with UC (**Figure 4A**), their mucosal immune profile was also dysregulated as it was characterised by a reduced relative frequency of 6 clusters (**Figure 4B**) and an increased relative frequency of 23 cell clusters (**Figure 4C**). Indeed, the inflamed UC mucosa, in agreement with that from colonic CD, also showed higher relative frequencies of T-cells (both memory CD4^+^ and non-classical CD4^-^CD8^-^), NK cells, NKT cells and IgG^+^ B-cells, together with a higher relative proportion of different APC subsets. Nonetheless, the circulating immune profile from UC patients displayed more differences referred to controls, than those from patients with CD, since it was characterised by a deficit of non-classical T-cells and monocytes (consistent with tissue-specific compartmentalisation of these population), and an increased relative frequency of several T-cell cluster (mainly of a memory and non-classical phenotype (**Figures 4D–F**).

**Figure 4:**
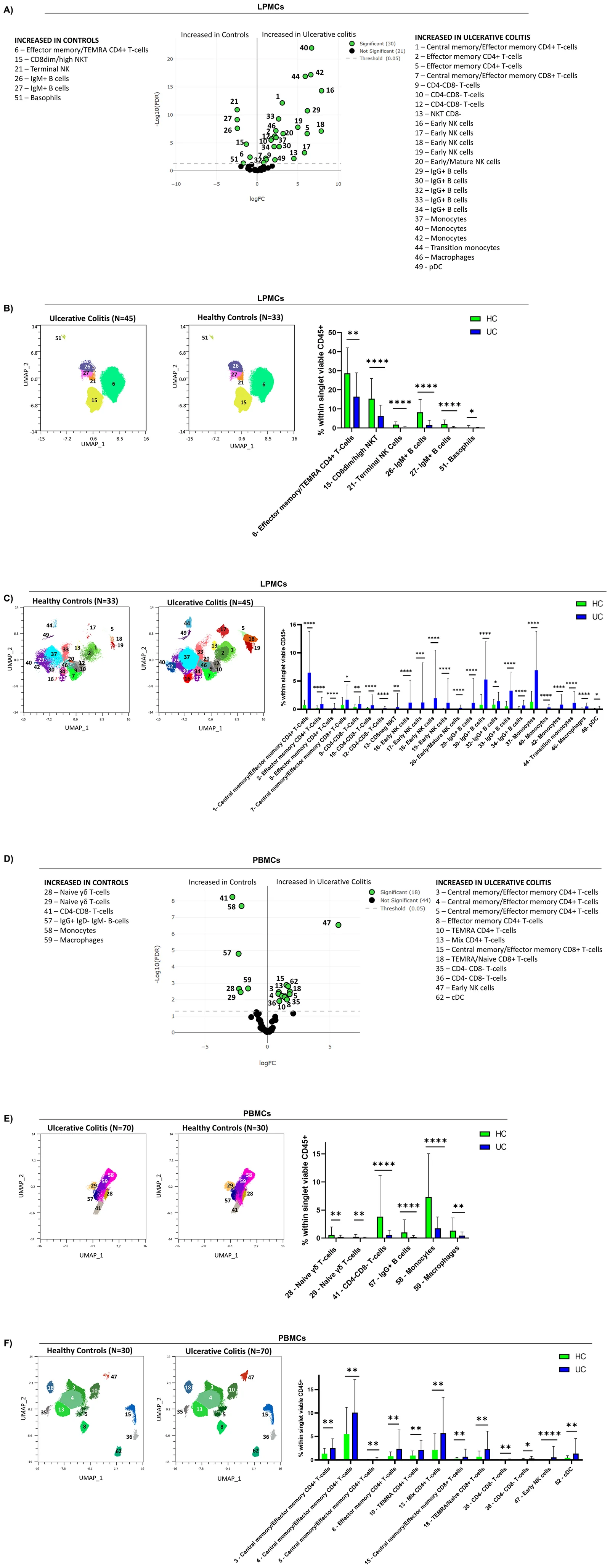
The mucosal and circulating ulcerative colitis immune fingerprint. **(A)** Volcano plot showing differential cluster abundance within the human lamina propria mononuclear cells (LPMCs) between patients with ulcerative colitis (UC) and controls. Each point represents a FlowSOM-defined cluster; the x-axis indicates log_2_ fold change between groups, and the y-axis shows statistical significance (–log_10_ FDR). Clusters reaching statistical significance after multiple-testing correction are highlighted. **(B)** Clusters significantly enriched in controls are projected onto the global UMAP representation, allowing visualization of their spatial distribution within the overall cellular landscape. Bar plots display the relative abundance of these clusters in each group. **(C)** Clusters significantly enriched in UC patients are projected onto the global UMAP, together with bar plots showing their relative abundance across groups. **(D)** As above, the same Volcano plot was also performed on the FlowSOM-defined clusters identified within peripheral blood mononuclear cells (PBMCs) from the same patients. **(E)** Clusters significantly enriched in healthy controls are projected onto the global UMAP representation, allowing visualization of their spatial distribution within the overall PBMCs landscape. Bar plots display the relative abundance of these clusters in each group. **(F)** Clusters significantly enriched in ulcerative colitis are projected onto the global UMAP, together with bar plots showing their relative abundance across groups. Differential abundance analysis was performed using edgeR, applying a generalised linear model with multiple-testing correction by the Benjamini–Hochberg FDR method in panels **(A, D)**. Unpaired two-tailed t-test was applied in panels **(B, C, E, F)**. P-value < 0.05 was considered as statistically significant (*p < 0.05; **p < 0.01; ***p < 0.001; ****p < 0.0001).

Given the similarities found between the mucosal and circulating immune profiles in both patients with UC and colonic CD, we next specifically addressed whether both IBD types displayed mucosal-specific immune signatures (**Figure 5A**). Indeed, and in agreement with the previously described immune profile, colonic CD was characterised by a further increased proportion of memory T-cells (**Figure 5B**), while the mucosal immune profile in UC was more mixed with a higher proportion of NK and B-cells (**Figure 5C**). Nevertheless, these differences were mainly abrogated in their circulating precursors (**Figures 5D–F**). Of note, plasmacytoid dendritic cell (pDC) were differentially expanded in the colonic compartment of both CD (**Figure 5B**) and UC (**Figure 5C**) patients, although this was not mirrored in blood.

**Figure 5:**
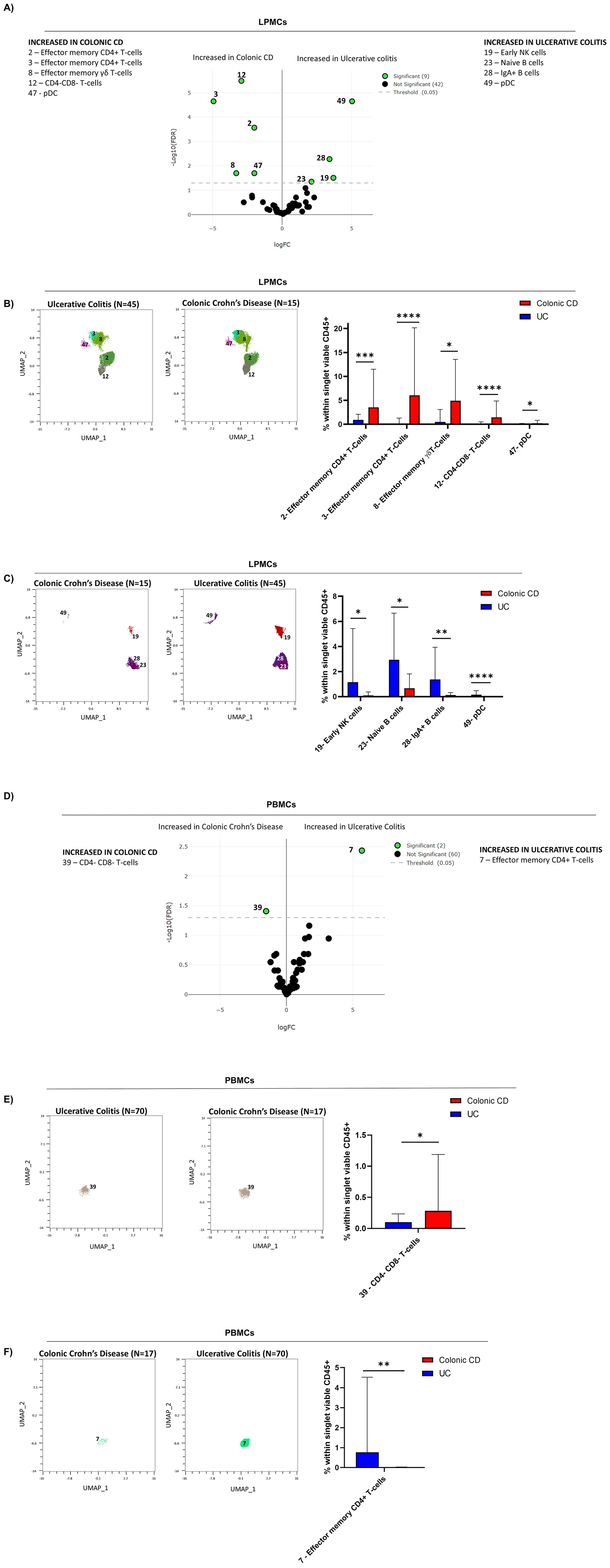
The differential immune profile between colonic Crohn´s disease and ulcerative colitis. **(A)** Volcano plot showing differential mucosal cluster abundance within lamina propria mononuclear cells (LPMCs) between patients with colonic Crohn’s disease (CD) and ulcerative colitis (UC). Each point represents one FlowSOM-defined cluster; the x-axis indicates log_2_ fold change between groups, and the y-axis shows statistical significance (–log_10_ FDR). Clusters reaching statistical significance after multiple-testing correction are highlighted. **(B)** Clusters significantly enriched in colonic CD are projected onto the global UMAP representation, allowing visualization of their spatial distribution within the overall cellular landscape. Bar plots display the relative abundance of these clusters in each group. **(C)** Clusters significantly enriched in UC are projected onto the global UMAP, together with bar plots showing their relative abundance across groups. **(D)** As above, the Volcano analysis was also performed within peripheral blood mononuclear cells (PBMCs). **(E)** Clusters significantly enriched in colonic CD are projected onto the global UMAP representation, allowing visualization of their spatial distribution within the overall PBMCs landscape. Bar plots display the relative abundance of these clusters in each group. **(F)** Clusters significantly enriched in UC are projected onto the global UMAP, together with bar plots showing their relative abundance across groups. Differential abundance analysis was performed using edgeR, applying a generalised linear model with multiple-testing correction by the Benjamini–Hochberg FDR method in panels **(A, D)**. An unpaired two-tailed t-test was applied in panels **(B, C, E, F)**. P-value < 0.05 was considered as statistically significant (*p < 0.05; **p < 0.01; ***p < 0.001; ****p < 0.0001).

Together, these results suggest that, although both colonic CD and UC display a dysregulated immune response referred to non-inflamed controls, colonic CD seems to display a more T-cell biased immune profile as opposed to patients with UC which have a mixed one. Indeed, the fact that these differences are mainly found in the tissue, but not in their circulating precursors, also suggests an increased capacity of the tissue to recruit circulating immune cells which are further conditioned in the mucosa to display and increased inflammatory profile.

### Lack of major differences between patients with ileal and colonic CD

Having described the similarities and differences between colonic CD and UC, and since the properties of the immune system systematically change through the length of the intestinal mucosa (25, 26), we next studied whether that is also translated into a differential immune response between patients with ileal and colonic CD. Our results revealed that patients with colonic CD had a specific increased relative abundance of some clusters engulfing effector memory CD4^+^ T-cells, NK cells, IgG+ B-cells and pDC (**Figures 6A, B**) coupled with increased relative abundance of circulating effector memory CD4^+^ T-cells in patients with ileal disease (**Figures 6C, D**) which nevertheless was not translated into the tissue. Hence, these results suggest that although patients with CD display an abnormal and dysregulated immune profile compared to controls (**Figure 2A**), the differences between patients with ileal and colonic manifestations are scarce.

**Figure 6:**
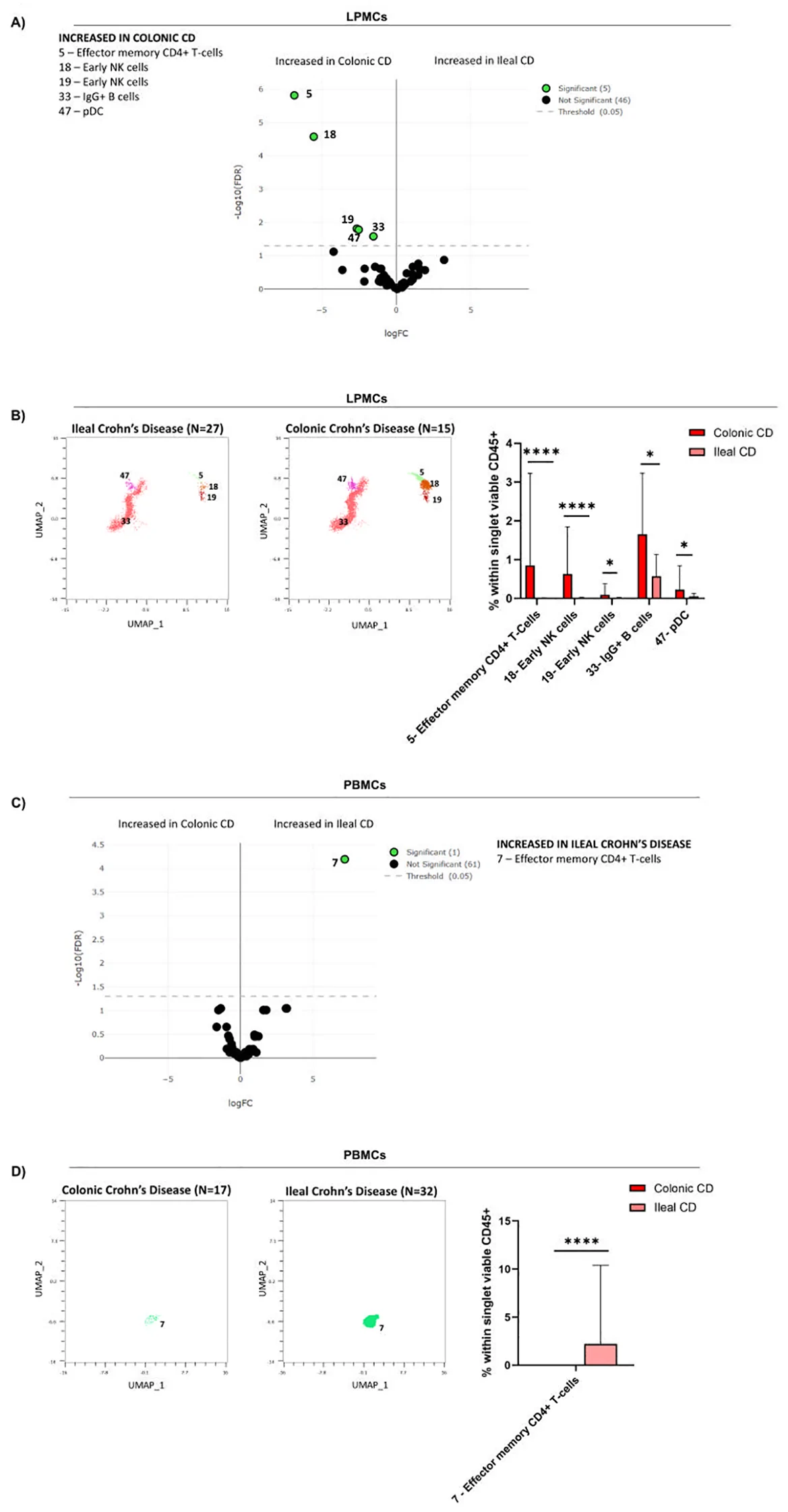
The immune profile between ileal and colonic Crohn´s disease. **(A)** Volcano plot showing differential cluster abundance, within lamina propria mononuclear cells (LPMCs) between patients with Crohn´s disease (CD) with either ileal or colonic disease. Each point represents one FlowSOM-defined cluster; the x-axis indicates log_2_ fold change between groups, and the y-axis shows statistical significance (–log_10_ FDR). Clusters reaching statistical significance after multiple-testing correction are highlighted. **(B)** Clusters significantly enriched in colonic CD are projected onto the global UMAP representation, allowing visualization of their spatial distribution within the overall cellular landscape. Bar plots display the relative abundance of these clusters in each group. **(C)** The same approach was performed on the circulating clusters identified within singlet viable CD45^+^ peripheral blood mononuclear cells (PBMCs). **(D)** Clusters significantly enriched in ileal CD are projected onto the global UMAP representation, allowing visualization of their spatial distribution within the overall PBMCs landscape. Bar plots display the relative abundance of these clusters in each group. Differential abundance analysis was performed using edgeR, applying a generalised linear model with multiple-testing correction by the Benjamini–Hochberg FDR method in panels **(A, C)**. An unpaired two-tailed t-test was applied in panels **(B, D)**. P-value < 0.05 was considered as statistically significant (*p < 0.05; **p < 0.01; ***p < 0.001; ****p < 0.0001).

### The biological sex is associated with the immune infiltrate of patients with UC and the circulating profile of patients with CD

We next studied the effect that the biological sex could elicit on the composition of the immune system. Our results revealed that, although the biological sex was not associated with the mucosal immune system from controls (**Supplementary Figure 2A**), and had a minor effect on that from patients with ileal CD (**Supplementary Figures 2B, C**) but not colonic CD (**Supplementary Figure 2D**), it nevertheless had an impact on the composition of the mucosal profile from patients with UC (**Figure 7A**). Hence female patients with endoscopically active UC displayed higher relative frequencies of effector memory T-cells as well as NK and NKT cells and IgG^+^ B-cells (**Figure 7B**).

**Figure 7:**
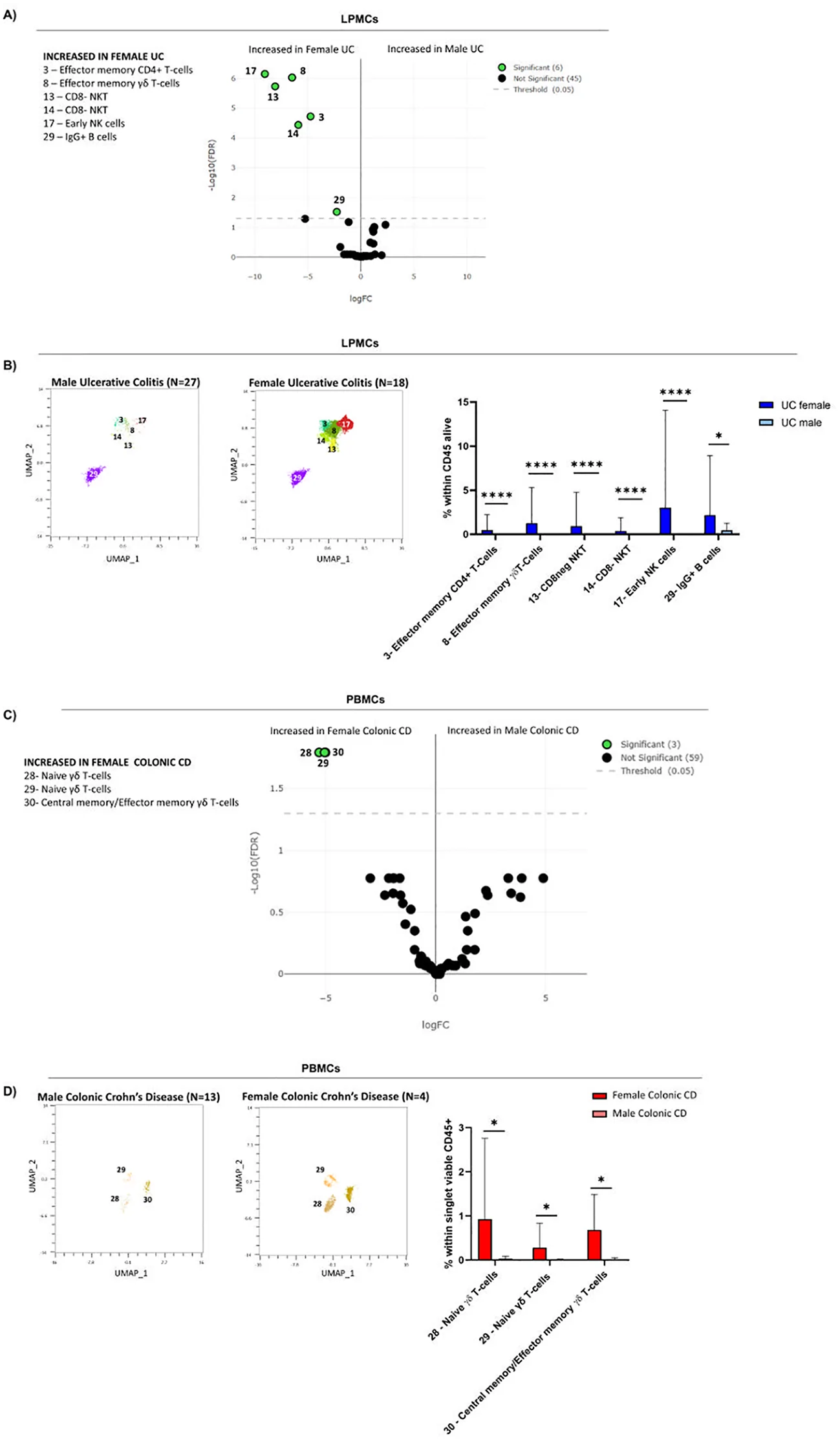
The sex perspective in ulcerative colitis. **(A)** Volcano plot showing differential cluster abundance within lamina propria mononuclear cells (LPMCs) between female and male patients with ulcerative colitis (UC). Each point represents one FlowSOM-defined cluster; the x-axis indicates log_2_ fold change between groups, and the y-axis shows statistical significance (–log_10_ FDR). Clusters reaching statistical significance after multiple-testing correction are highlighted. **(B)** Clusters significantly enriched in female patients are projected onto the global UMAP representation, allowing visualization of their spatial distribution within the overall cellular landscape. Bar plots display the relative abundance of these clusters in each sex. **(C)** The same Volcano approach was also applied within peripheral blood mononuclear cells (PBMCs). **(D)** Clusters significantly enriched in female patients are projected onto the global UMAP representation, allowing visualization of their spatial distribution within the overall PBMCs landscape. Bar plots display the relative abundance of these clusters in each sex. Differential abundance analysis was performed using edgeR, applying a generalised linear model with multiple-testing correction by the Benjamini–Hochberg FDR method in panels **(A, C)**. An unpaired two-tailed t-test was applied in panels **(B, D)**. P-value < 0.05 was considered as statistically significant (*p < 0.05; **p < 0.01; ***p < 0.001; ****p < 0.0001).

As for the circulating immune system, and although again the biological sex had no effect in controls (**Supplementary Figure 2E**), in patients with colonic CD (but not in those with ileal disease; **Supplementary Figure 2F**) it was associated with increased relative abundance of different clusters of circulating Tcells (**Figures 7C, D**) with nevertheless little effect on the immune system of patient with UC (**Supplementary Figures 2G, H**).

### Age is associated with the immune response in UC but not in CD

We also studied whether the immune response was modulated by the age of the patients. In this regard, and given that the mean age of CD patients was 47.8, while in UC it was 50.1 (**Table 1**), an arbitrary threshold of 50 years was considered within each cohort to compare younger and more adult patients (**Supplementary Tables 2**, **3**).

As with the biological sex, the age had none or little effect over the mucosal immune system composition from controls (**Supplementary Figure 3A**) or patients with CD, either with ileal (**Supplementary Figures 3B, C**) or colonic disease (**Supplementary Figure 3D**). Nevertheless, the age also affected the UC colonic immune infiltrate (**Figure 8A**) since elderly patients displayed increased proportion of NKT and NK clusters (**Figure 8B**) coupled with a deficit of different T-cell, NK and basophil clusters (**Figure 8C**). Moreover, the age also had an impact of their circulating precursors (**Figure 8D**) with increased relative abundance of a NKT cluster in patients over 50 (**Figure 8E**) and a depletion of two clusters of CD4^-^CD8^-^ T-cells in younger patients (**Figure 8F**). Nevertheless, the age of the patients did not have any impact of the circulating immune system from controls (**Supplementary Figure 3E**) or patient with ileal (**Supplementary Figure 3F**) or colonic CD (**Supplementary Figure 3G**). Together, these results highlight that although biological sex had an impact on the mucosal immune system from patients with UC and the circulating one from patients with CD, when it comes to the age it actually has an effect on the patients with UC (both at the mucosal and the circulating level) but not with CD.

**Figure 8:**
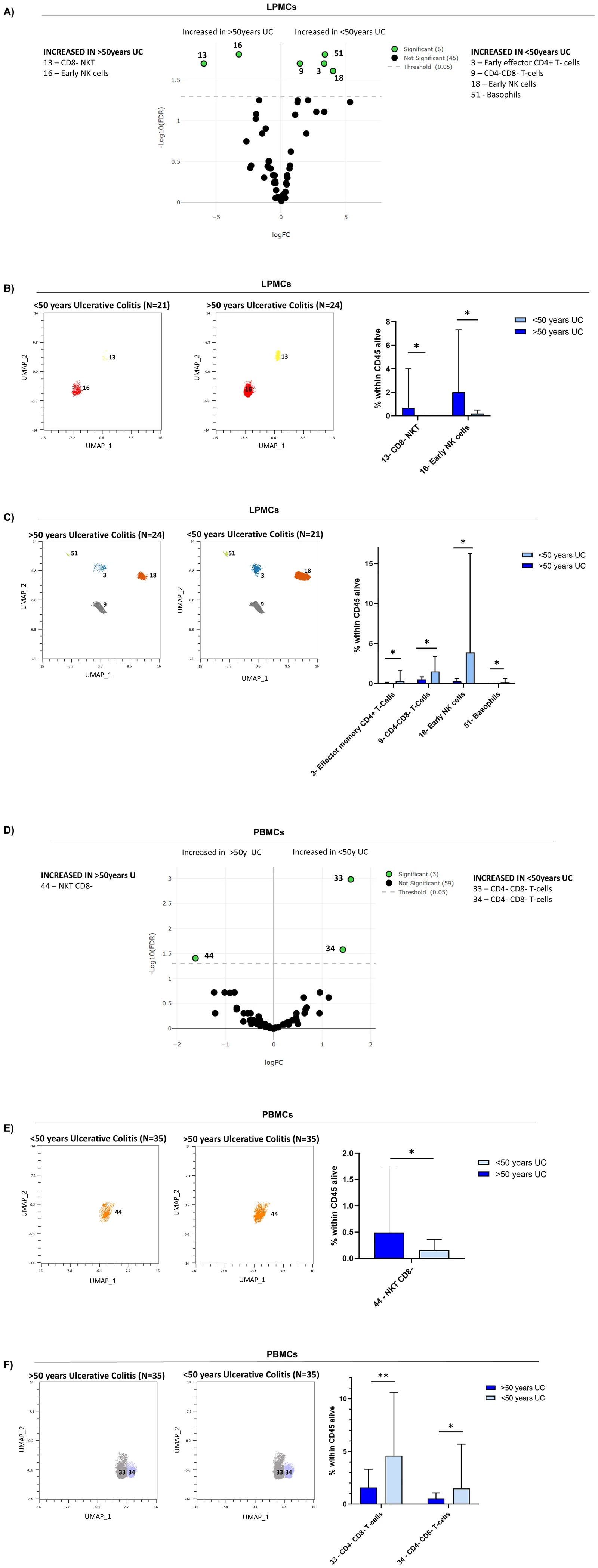
The age effects the immune response in patients with ulcerative colitis. **(A)** Volcano plot showing differential cluster abundance within lamina propria mononuclear cells (LPMCs) between patients with Ulcerative Colitis (UC) aged >50 years and ≤50 years. Each point represents one FlowSOM-defined cluster; the x-axis indicates log_2_ fold change between age groups, and the y-axis shows statistical significance (–log_10_ FDR). Clusters reaching statistical significance after multiple-testing correction are highlighted. **(B)** Clusters significantly enriched in patients aged >50 years are projected onto the global UMAP representation, allowing visualization of their spatial distribution within the overall cellular landscape. Bar plots display the relative abundance of these clusters across age groups. **(C)** Clusters significantly enriched in patients aged ≤50 years are projected onto the global UMAP representation, together with bar plots showing their relative abundance across age groups. **(D)** The Volcano approach was performed within peripheral blood mononuclear cells (PBMCs). **(E)** Clusters significantly enriched in patients aged >50 years are projected onto the global UMAP representation, allowing visualization of their spatial distribution within the overall PBMCs landscape. Bar plots display the relative abundance of these clusters across age groups. **(F)** Clusters significantly enriched in patients aged ≤50 years are projected onto the global UMAP representation, together with bar plots showing their relative abundance across age groups. Differential abundance analysis was performed using edgeR, applying a generalised linear model with multiple-testing correction by the Benjamini–Hochberg FDR method in panels **(A, D)**. Unpaired two-tailed t-test was applied in panels **(B, C, E, F)**. P-value < 0.05 was considered as statistically significant (*p < 0.05; **p < 0.01; ***p < 0.001; ****p < 0.0001).

### The immunome from UC patients, but not from those with CD, is also influenced by disease severity

Finally, and within both CD and UC, we further studied whether endoscopic disease severity had an impact on the composition of the immune system both in patients with UC and ileal CD (patients with colonic CD were excluded from this analysis since there were no major differences in disease severity in this cohort so the analysis could not be performed).

Our results revealed that disease severity also had little impact on the composition of the mucosal (**Supplementary Figures 4A, B**) and circulating (**Supplementary Figure 4C**) immune system from patients with ileal CD, as opposed to patients with UC (**Figure 9A**). Indeed, the clusters that had been previously described to be expanded in the colon from patients with UC (including effector memory T-cells, non-classical CD4^-^CD8^-^ T-cells, NK and NKT cells and monocytes), had their proportion further increased in patients with severe disease (**Figure 9B**), while among two clusters of mucosal monocytes and pDC whose proportion was increased in patients with mild disease (**Figure 9C**), the latter also had an increased proportion in the blood from these patients (**Supplementary Figures 4D, E**).

**Figure 9:**
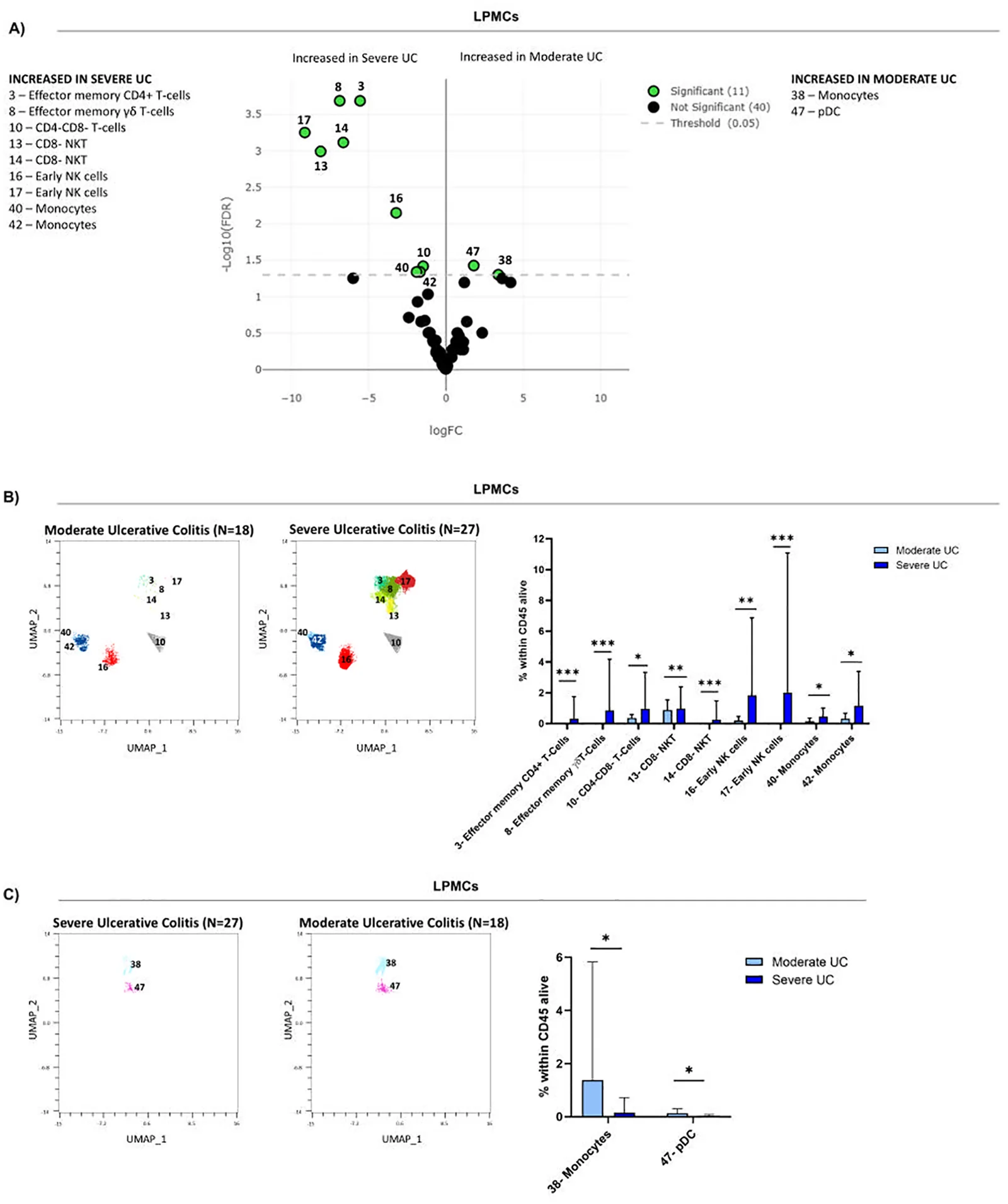
Disease severity–associated immune clusters in ulcerative colitis. **(A)** Volcano plot showing differential cluster abundance, within lamina propria mononuclear cells (LPMCs) between patients with moderate and severe ulcerative colitis. Each point represents one FlowSOM-defined cluster; the x-axis indicates log_2_ fold change between severity groups, and the y-axis shows statistical significance (–log_10_ FDR). Clusters reaching statistical significance after multiple-testing correction are highlighted. **(B)** Clusters significantly enriched in patients with severe ulcerative colitis are projected onto the global UMAP representation, allowing visualization of their spatial distribution within the overall cellular landscape. Bar plots display the relative abundance of these clusters across severity groups. **(C)** Clusters significantly enriched in patients with moderate ulcerative colitis are projected onto the global UMAP representation, together with bar plots showing their relative abundance across severity groups. Differential abundance analysis was performed using edgeR, applying a generalised linear model with multiple-testing correction by the Benjamini–Hochberg FDR method in panel **(A)**. Unpaired two-tailed t-test was applied in panels **(B, C)**. P-value < 0.05 was considered as statistically significant (*p < 0.05; **p < 0.01; ***p < 0.001; ****p < 0.0001).

## Discussion

IBD pathogenesis results from interactions between genetic susceptibility, environmental exposures, microbial composition, and immune dysregulation, converging on an aberrant mucosal immune system activation (27, 28). Although systemic immune alterations may mirror intestinal inflammation (29), understanding how immune perturbations diverge between CD, UC, and controls remains essential for identifying disease mechanisms, potential biomarkers and therapeutic targets.

Over the past decade, substantial advances have been made in identifying immune cell subsets associated with IBD through a variety of omics-based approaches (30–32). In this context, our study provides a comprehensive, high-dimensional immunophenotypic characterization of leukocyte subsets in both peripheral blood and intestinal tissue, using an adapted OMIP-69 spectral cytometry panel (22).

A principal observation is that immune alterations distinguishing IBD were more pronounced in intestinal tissue than in blood. The mucosal immune profile exhibited broad shifts across immune populations, whereas peripheral blood showed limited differences, supporting IBD as a predominantly mucosa-driven disease in which blood only partially reflects gut inflammation (33, 34). This agrees with previous immune-profiling studies showing partial concordance between mucosal and circulating compartments (35–38). These findings have implications for biomarker development, as blood signatures may miss disease-relevant tissue-resident changes. Although blood sampling remains advantageous due to its accessibility and suitability for longitudinal monitoring, this divergence underscores the limited sensitivity of blood-based biomarkers to fully reflect intestinal inflammation and stresses the need for tissue-resolved profiling in mechanistic and translational research. Comparisons with controls should nevertheless be interpreted considering that control samples were obtained from individuals undergoing colonoscopy for colorectal cancer screening, anaemia or abdominal pain evaluation, all of whom lacked endoscopic or histological inflammation and had no known malignant, autoimmune or inflammatory disease. Their demographic characteristics are provided in **Supplementary Tables 2** and **3** and did not show relevant differences compared with the patient groups.

Another central finding of our study is the lack of major differences between colonic and ileal CD. Although ileum and colon differ in microbial exposure, epithelial architecture, immune cell composition, and lymphoid tissue organisation (39, 40), and ileal and colonic CD are considered distinct subtypes (41, 42), mucosal and circulating immune profiles were broadly consistent (**Figure 6**). This comparison should be interpreted considering the anatomical origin of the samples, as ileal CD biopsies were obtained from the terminal ileum whereas colonic CD and control samples were obtained from the colon. Thus, disease-location-associated differences may partly reflect regional immune specialisation of the sampled intestinal segment. Nevertheless, the altered immune populations identified in ileal and colonic CD were broadly overlapping, supporting the concept that these CD phenotypes shared a similar compositional immune profile in this cohort. Previously reported transcriptome differences between ileal and colonic CD (43) may therefore reflect tissue-specific immune function rather than distinct immune-cell composition.

Of note, another striking pattern emerging from our data is that UC displays substantially greater immunological heterogeneity than CD when stratified by demographic and clinical variables. Biological sex modulated the mucosal immune infiltrate in UC but not in CD, with female patients displaying an expansion of effector memory T cells, NK, NKT cells and IgG^+^ B-cells in the colonic mucosa (**Figures 7A, B**); consistent with biological sex-dependent inflammatory niches in UC (44). Similarly, endoscopic severity also induces a clear compositional shift in UC mucosa (**Figure 9**), in line with single-cell studies distinguishing active and inactive UC more consistently than CD (30, 45). Age further modulated UC immune response, with differential NK and NKT cell populations in tissue and blood (**Figures 8A–F**). This susceptibility may reflect the continuous, superficial mucosal inflammation of UC, whereas transmural and discontinuous CD inflammation may impose a more uniform inflammatory programme. Collectively, these findings highlight UC as an immunologically heterogeneous disease in which patient subgroup stratification may be particularly important for both biomarker discovery and therapeutic decision-making.

These stratified findings should be interpreted in light of potential clinical and methodological limitations. The BioIBD cohort represents a real-world population of patients with active IBD sampled before initiation of a first administration of biological therapy with a given mechanism of action, thereby reducing the direct impact of this therapy on baseline immune profiles. This clinically representative baseline setting is a valuable feature of the cohort, as it captures immune-cell composition in active disease before exposure to the therapeutic mechanism being initiated. Nevertheless, age, biological sex and endoscopic severity may covary with disease duration, smoking status, disease phenotype and previous or concomitant treatment exposure. Treatment-adjusted sensitivity and fully adjusted multivariable analyses were not performed because of the reduced effective sample size after subgrouping. Accordingly, subgroup-associated immune differences should be interpreted as hypothesis-generating immune patterns rather than definitive evidence of independent demographic or clinical effects.

Although the overall cohort size was appropriate for high-dimensional cytometry profiling, effective sample size decreased after stratification by disease subtype, location, biological sex, age and severity. This may affect the statistical power and stability of some cluster-level associations. The age cutoff of 50 years was selected because it closely approximated the median age of the study population, allowing comparison of younger and older adult groups while preserving subgroup size. Nevertheless, modelling age as a continuous variable in large cohorts would provide a more refined assessment. Finally, the absence of an independent validation cohort means that subgroup-associated immune patterns should be considered hypothesis-generating and require external validation.

Together, our results expand the understanding of immune dysregulation in IBD by providing a high-resolution map of leukocyte heterogeneity across tissues and patient subgroups, providing a framework for future studies assessing whether patient-specific immune fingerprints can inform biomarker development and treatment stratification.

## Data Availability

The raw data supporting the conclusions of this article will be made available by the authors, without undue reservation.
