# Supplementary material for: Ulcerative colitis, but not Crohn’s disease, displays demographically and clinically driven mucosal immune heterogeneity: implications for patient stratification
Source: Front Immunol. 2026 Sep 24;17:1965046. doi: 10.3389/fimmu.2026.1965046 (PMC13646131; doi:10.3389/fimmu.2026.1965046)
Supplement: Supplementary file 1 [file Table1.docx]

**SUPPLEMENTARY TABLES**

**Supplementary Table 1: Antibody panel used for spectral flow cytometry immunophenotyping.** List of monoclonal antibodies included in the spectral flow cytometry panel, indicating antigen specificity, fluorochrome, antibody clone, and manufacturer. This panel was designed to enable comprehensive immunophenotyping of both peripheral blood and lamina propria mononuclear cells for the identification of major innate and adaptive immune cell populations relevant to inflammatory bowel disease. All antibodies were used for surface staining.

| **Specifity** | **Fluorochrome** | **Clone** | **Company** |
| --- | --- | --- | --- |
| CCR7 | BUV805 | 3D12 | BD |
| CD3 | BV510 | SK7 | BioLegend |
| CD4 | cFluorYG584 | SK3 | Cytek |
| CD8 | cFluor B532 | SK1 | Cytek |
| CD11c | eFluor 450 | 3.9 | ebioscience |
| CD14 | Spark Blue 550 | 63D3 | BioLegend |
| CD16 | BUV 496 | 3G8 | BD |
| CD19 | cFluor BYG750 | HIB19 | Cytek |
| CD20 | cFluor V547 | 2H7 | Cytek |
| CD24 | PE-Alexa 610 | SN3 | Thermo Fisher |
| CD25 | BV785 | BC96 | BioLegend |
| CD28 | BV650 | CD28.2 | BioLegend |
| CD38 | APC/Fire810 | HIT2 | BioLegend |
| CD39 | BUV661 | TU66 | BD |
| CD45 | PerCP | HI30 | BioLegend |
| CD45RA | BUV395 | 5H9 | BD |
| CD56 | BUV 737 | NCAM16.2 | BD |
| CD79a (IgA) | BV421 | HM47 | BD |
| CD95 (Fas) | PE-Cy5 | DX2 | BioLegend |
| CD123 | Super Bright 436 | 6H6 | ebioscience |
| CD127 | APC-R700 | HIL-7R-M21 | BD |
| CXCR3 | BUV615 | 1C6/CXCR3 | BD |
| CXCR5 | BV750 | RF8B2 | BD |
| HLA-DR | PE Fire 810 | L243 | BioLegend |
| IgD | BV480 | IA6-2 | BD |
| IgG | BV605 | G18-145 | BD |
| IgM | BV570 | MHM-88 | BioLegend |
| TCR γδ | cFluor BYG710 | B1 | Cytek |
| Viability | Live/Dead UV Blue | - | Invitrogen |

**Supplementary Table 2: Baseline characteristics of LPMC samples after quality control filtering.** Demographic and clinical characteristics of subjects included in the LPMC analysis after quality control filtering. Only samples with ≥5,000 viable leukocyte events within the singlet viable cell gate were retained for downstream analyses. Continuous variables are reported as mean (range), and categorical variables as number (percentage). Disease-specific clinical characteristics are presented separately for healthy controls (HC), Crohn’s disease (CD), and ulcerative colitis (UC).

| **Characteristics** | **Healthy controls (HC)**  **(n=33)** | **Crohn’s Disease (CD) (n=42)** | **Ulcerative colitis (UC) (n=45)** |
| --- | --- | --- | --- |
| **Female sex, n (%)** | 18 (54.5) | 19 (45.2) | 18 (40) |
| **Male sex, n (%)** | 15 (45.5) | 23 (54.8) | 27 (60) |
| **Age, years, mean (range)** | 57 (31-75) | 50 (26-74) | 52 (22-78) |
| <50years, n (%) | 10 (30.3) | 23 (54.8) | 21 (46.7) |
| >50years, n (%) | 23 (69.7) | 19 (45.2) | 24 (53.3) |
| **Location** |  | | |
| Ileal, n (%) | 0 (0) | 27 (64.3) | 0 (0) |
| Colonic, n (%) | 33 (100) | 15 (35.7) | 45 (100) |
| **CD endoscopic severity**  **(SES-CD)** |  | | |
| Mild 3-6, n (%) |  | 10 (23.8) |  |
| Moderate 7-15, n (%) |  | 28 (66.7) |  |
| Severe ≥16, n (%) |  | 4 (9.5) |  |
| **UC endoscopic severity**  **(Mayo score)** |  | | |
| Moderate (Mayo 2), n (%) |  |  | 18 (40) |
| Severe (Mayo 3), n (%) |  |  | 27 (60) |

**Supplementary Table 3: Baseline characteristics of PBMC samples after quality control filtering.** Demographic and clinical characteristics of subjects included in the LPMC analysis after quality control filtering. Only samples with ≥10,000 viable leukocyte events within the singlet viable cell gate were retained for downstream analyses. Continuous variables are reported as mean (range), and categorical variables as number (percentage). Disease-specific clinical characteristics are presented separately for healthy controls (HC), Crohn’s disease (CD), and ulcerative colitis (UC).

| **Characteristics** | **Healthy controls (HC)**  **(n=30)** | **Crohn’s Disease (CD) (n=49)** | **Ulcerative colitis (UC) (n=70)** |
| --- | --- | --- | --- |
| **Female sex, n (%)** | 15 (50) | 19 (38.8) | 30 (42.9) |
| **Male sex, n (%)** | 15 (50) | 30 (61.2) | 40 (57.1) |
| **Age, years, mean (range)** | 55 (34-76) | 50 (23-75) | 50 (19-81) |
| <50years, n (%) | 8 (26.7) | 26 (53.1) | 35 (50) |
| >50years, n (%) | 22 (73.3) | 23 (46.9) | 35 (50) |
| **Location** |  | | |
| Ileal, n (%) | 0 (0) | 32 (65.3) | 0 (0) |
| Colonic, n (%) | 30 (100) | 17 (34.7) | 70 (100) |
| **CD endoscopic severity (SES-CD)** |  | | |
| Mild 3-6, n (%) |  | 17 (34.7) |  |
| Moderate 7-15, n (%) |  | 26 (53.1) |  |
| Severe ≥16, n (%) |  | 6 (12.2) |  |
| **UC endoscopic severity (Mayo score)** |  | | |
| Moderate (Mayo 2), n (%) |  |  | 26 (37.1) |
| Severe (Mayo 3), n (%) |  |  | 44 (62.9) |

**Supplementary Table 4: Cell cluster identification of total lamina propria mononuclear cells.** For each of the 51 identified FlowSOM clusters within total lamina propria mononuclear cell, the cell population and subset to which it belongs is shown, alongside the specific lineage and functional markers that they express.

| **ID** | **Population** | **Subset** | **Phenotypic expression** | **Functional expression** |
| --- | --- | --- | --- | --- |
| 1 | **CD4+ Tcells** | **Central memory/ Effector memory** | CD3 , CD4 , CD39 , CD2 , CD28 , HLADR | CD95 |
| 2 | **CD4+ T-cells** | **Effector memory** | CD3, CD4 , CD14 , CD11c , CD39 , IgG , CD27 | CD95 |
| 3 | **CD4+ Tcells** | **Effector memory** | CD3 , CD20 , HLADR , CD4 , CD14 , CD16 , CD11c , CD39 , IgG , CD141 , CD25 , CD28 , CD38 , CD27 | CD95 |
| 4 | **CD4+/Tgd Tcells** | **Effector memory** | CD3 , TCRγδ , CD20 , HLADR , CD4 , CD8dim , CD14 , CD11c , IgG , CD24 , CD25 , CD28 |  |
| 5 | **CD4+ T-cells** | **Effector memory** | CD3 , CD123 , CD4 , CD14 , IgG , CD141 , CD2 , CD24 , CD28 , CD39 | CXCR3 , CD95 |
| 6 | **CD4+ Tcells** | **Effector memory/TEMRA** | CD3 , CD4 , CD127 | CD95 |
| 7 | **CD8+ Tcells** | **Central memory/ Effector memory** | CD3 , HLADR , CD8 , CD39 , CD2 , CD24 | CD95 |
| 8 | **Tγδ T-cells** | **Effector memory** | CD3 , CD20 , HLADR , CD14 , CD11c , CD39 , IgG , CD141 | CD95 |
| 9 | **CD4- CD8- T Cells** |  | CD3 , HLADR | CD95 |
| 10 | **CD4- CD8- T Cells** |  | CD3 , HLADR |  |
| 11 | **CD4- CD8- T Cells** |  | CD3 , CD127 | CD95 |
| 12 | **CD4- CD8- T Cells** |  | CD3 , CD14 | CXCR5 , CD95 |
| 13 | **NKT** | **CD8-** | CD3 , CD19 , CD56 , CD14 , CD11c , CD39 , IgG , CD141 , CD27 , CD38 , CD24 | CD95 |
| 14 | **NKT** | **CD8-** | CD3 , TCRγδ , CD20 , CD19 , CD56 , HLADR , CD4 , CD14 , CD16 , CD11c , CD39 , IgG , CD24 , CD28 | CD95 |
| 15 | **NKT** | **CD8dim/high** | CD3 , CD8 | CD95 |
| 16 | **NK cells** | **Early** | CD3 , CD20 , CD14 , CD16 , CD39 , IgG , CD2 , CD24 , CD25 | CD95 |
| 17 | **NK cells** | **Early** | CD3, CD14, CD16 , CD11c , IgG , CD27 | CD95 |
| 18 | **NK cells** | **Early** | CD4 , CD16 , CD39 , IgG , CD24 , CD28 , CD123 |  |
| 19 | **NK cells** | **Early** | CD4 , CD16 , CD24 , CD28 , CD123 , CD39 |  |
| 20 | **NK cells** | **Early-Mature** | CD16 , CD45RA , CD56 |  |
| 21 | **NK cells** | **Terminal** | CD56 , CXCR5 , CD38 | CXCR5 , CD95 |
| 22 | **B cells** | **Naive** | CD20 , CD19 , CD56 , HLADR , CD4 , CD14 , CD16 , CD11c , CD39 , IgD , CD24 , CD27 , CD28 | CD95 |
| 23 | **B cells** | **Naive** | CD20 , CD19 , HLADR , CD45RA , IgD , IgM , IgG , CD39 | CXCR5 |
| 24 | **B cells** | **IgM+** | CD20 , CD19 , HLADR , CD45RA , CD39 , IgA | CXCR5 |
| 25 | **B cells** | **IgM+** | CD20 , CD19 , HLADR , CD45RA , CD39 , IgM , CD24 , CD1c | CXCR5 |
| 26 | **B cells** | **IgM+** | CD39 , CD38 | CD95 |
| 27 | **B cells** | **IgM+** | CD39 , CD38 , CD56 | CD95 |
| 28 | **IgM- IgD- B cells** | **IgA+** | CD3 , CD20 , CD19 , HLADR , CD11c , CD45RA , CD39 ,IgA, IgD , IgG , CD24 | CXCR5 , CD95 |
| 29 | **IgM- IgD- B cells** | **IgG+** | CD3, CD20 , HLADR , CD14, CD16 , CD11c , CD39 , IgG , CD24 , CD25 , CD38 , CD141 |  |
| 30 | **IgM- IgD- B cells** | **IgG+** | CD3 , CD20 , HLADR , CD14 , CD16 , CD11c , CD39 , IgG , CD24 , CD38 , CD141 | CD95 |
| 31 | **IgM- IgD- B cells** | **IgG+** | CD3 , CD19 , CD56 , HLADR , CD14 , CD16 , CD39 , IgG , CD141 , CD25 , CD27 , CD38 |  |
| 32 | **IgM- IgD- B cells** | **IgG+** | CD3 , CD20 , CD19 , HLADR , CD14 , CD45RA , CD39 , IgG | CD95 |
| 33 | **IgM- IgD- B cells** | **IgG+** | CD3 , CD20 , CD19 , HLADR , CD14 , CD11c , CD39 , IgG , CD24 , CD38 , CD45RA |  |
| 34 | **IgM- IgD- B cells** | **IgG+** | CD3 , CD20 , CD19 , HLADR , CD14 , CD11c , CD45RA , IgG , CD24 | CXCR5 , CD95 |
| 35 | **IgM- IgD- B cells** | **IgG+** | CD3 , CD20 , CD19 , HLADR , CD14 , CD11c , CD39 , IgG , IgA, CD141 , CD24 , CD56 | CXCR5 , CD95 |
| 36 | **IgM- IgD- B cells** | **IgG+** | CD20 , CD19 , CD8 , CD16 , CD39 , IgD , IgG , CD24 , CD25 , CD141 | CXCR5 , |
| 37 | **Monocytes** |  | CD3 , HLADR , CD14 , CD11c , CD39 , IgG , CD24 | CD95 , CXCR5 |
| 38 | **Monocytes** |  | CD3 , HLADR , CD14 , CD11c , CD39 , IgG , CD141 , CD45RA | CD95 , |
| 39 | **Monocytes** |  | CD3 , CD20 , HLADR , CD14 , CD11c , CD39 , IgG , CD24 | CXCR5 , CD95 |
| 40 | **Monocytes** |  | CD20 , CD56 , HLADR , CD14 , CD16 , CD11c , IgG , CD141 , CD24 , CD1c | CD95 |
| 41 | **Monocytes** |  | CD3 , CD20 , CD56 , HLADR , CD14 , CD16 , CD11c , CD39 , IgG , CD141 , CD24 , CD28 | CD95 |
| 42 | **Monocytes** |  | CD3 , CD20 , HLADR , CD14 , CD16 , CD11c , IgD , IgG , CD141 , CD24 , CD1c , CD56 , CD28 | CD95 |
| 43 | **Monocytes** | **Transition** | CD3 , CD20 , HLADR , CD14 , CD16 , CD11c , CD39 , IgG , CD24 | CD95 |
| 44 | **Monocytes** | **Transition** | CD20 , CD56 , HLADR , CD14 , CD11c , CD39 , IgG , CD127 | CD95 |
| 45 | **Monocytes** | **Transition** | CD3 , CD56 , HLADR , CD14 , CD11c , CD39 , IgG | CD95 |
| 46 | **Macrophages** |  | CD3 , CD20 , HLADR , CD14 , CD39 , IgG , CD24 | CD95 |
| 47 | **pDC** |  | TCRγδ , CD19 , HLADR , CD123 , CD4 , CD8 , CD16 , CCR7 , CD39 , IgD , IgM , CD127 , CD24 , CD25 , CD38 | CXCR3 , CD95 |
| 48 | **pDC** |  | TCRγδ , HLADR , CD4 , CD8 , CD16 , CD39 , IgD , IgG , CD24 , CD25 , CD28 , CD127 | CD95 |
| 49 | **pDC** |  | CD56 , HLADR , CD123 , CD8 , CD16 , CCR7 , CD39 , IgD , IgG , CD127 , CD24 , CD25 , CD1c | CXCR5 , CD95 |
| 50 | **cDC** |  | CD20 , HLADR , CD11c , CD39 , IgG , CD27 | CD95 |
| 51 | **Basophils** |  | CD56 , CD123 , CD4 , CD8 , CD16 , CD11c , CCR7 , CD127 , CD24 , IgA | CXCR3 , CXCR5 , CD95 |

**Supplementary Table 5: Cell cluster identification of total peripheral blood mononuclear cells.** For each of the 62 identified FlowSOM clusters within total peripheral blood mononuclear cell, the cell population and subset to which it belongs is shown, alongside the specific lineage and functional markers that they express.

| **ID** | **Population** | **Subset** | **Phenotypic expression** | **Functional expression** |
| --- | --- | --- | --- | --- |
| 1 | **CD4+ T-cells** | **Central memory** | CD3 , HLADR , CD14 , CD39 , IgM , IgG , CD141 , CD38 , CD4 | CD95 |
| 2 | **CD4+ T-cells** | **Central memory** | CD3 , HLADR , CD4 , CD14 , CD39 , IgG , CD141 , CD127 , CD38 | CD95 |
| 3 | **CD4+ T-cells** | **Central memory- Effector memory** | CD3 , CD4 , CD2 , CD28 , HLADR | CD95 |
| 4 | **CD4+ T-cells** | **Central memory- Effector memory** | CD3 , CD4 , CD127 , CD2 , CD28 | CD95 |
| 5 | **CD4+ T-cells** | **Central memory-Effector memory** | CD3 , CD20 , CD4 , CD16 , CD39 , CD127 , CD24 , CD45RA | CD95 |
| 6 | **CD4+ T-cells** | **Effector memory** | CD3 , HLADR , CD4 , CD39 , CD2 , CD28 , CD20 , IgG | CD95 |
| 7 | **CD4+ T-cells** | **Effector memory** | CD3 , HLADR , CD14 , CD11c , CD39 , IgM , IgG , CD127 , CD141 , CD38 , CD4 | CXCR3 , CD95 |
| 8 | **CD4+ T-cells** | **Effector memory** | CD3 , HLADR , CD14 , CD11c , CD39 , IgM , IgG , CD141 , CD38 , CD4 | CXCR3 , CD95 |
| 9 | **CD4+ T-cells** | **Effector memory-TEMRA** | CD3 , CD4 , CD2 | CD95 |
| 10 | **CD4+ T-cells** | **TEMRA** | CD3 , CD20 , CD19 , HLADR , CD4 , CD45RA , CD39 , IgG | CXCR3 , CXCR5 , CD95 |
| 11 | **CD4+ T-cells** | **TEMRA-Naive** | CD3 , CD4 , CD16 , CD45RA , CD127 , CD2 , CD27 , CD28 , CD20 | CD95 |
| 12 | **CD4+ T-cells** | **TEMRA-Naive** | CD3 , CD20 , CD19 , HLADR , CD4 , CD45RA , CD39 , IgD , IgM , IgA, CD127 , CD24 , CD27 | CXCR5 , CD95 |
| 13 | **CD4+ T-cells** | **Mix** | CD3 , CD4 , CD45RA |  |
| 14 | **CD4+ T-cells** | **Mix** | CD3 , CD20 , HLADR , CD4 , CD14 , CD11c , CD45RA , CD39 , IgM , CD127 , CD141 , CD2 , CD24 , CD25 , CD27 , CD28 | CXCR3 , CXCR5 , CD95 |
| 15 | **CD8+ T-cells** | **Central memory- Effector memory** | CD3 , HLADR , CD8 , CD2 , CD38 , CD20 | CD95 |
| 16 | **CD8+ T-cells** | **Effector memory** | CD3 , CD8 , CD2 | CD95 |
| 17 | **CD8+ T-cells** | **TEMRA** | CD3 , CD8 , CD45RA , CD2 | CD95 |
| 18 | **CD8+ T-cells** | **TEMRA-Naive** | CD3 , CD8 , CD45RA |  |
| 19 | **CD8+ T-cells** | **TEMRA-Naive** | CD3 , CD56 , CD8 , CD16 , CD45RA , CD39 , CD127 , CD2 , CD38 , CD20 | CD95 |
| 20 | **CD8+ T-cells** | **Naive** | CD3 , CD20 , CD8 , CD16 , CD39 , CD127 , CD2 , CD27 , CD38 , CD56 , HLADR , CD14 | CXCR3 , CD95 |
| 21 | **CD8+ T-cells** | **Naive** | CD3 , CD20 , CD19 , HLADR , CD8 , CD45RA , CD39 , IgD , IgM , IgA CD127 , CD24 , CD27 | CXCR5 , CD95 |
| 22 | **CD8+ T-cells** | **Mix** | CD3 , CD8 , CD127 , CD2 | CD95 |
| 23 | **γδ T-cells** | **Central Memory** | CD3 , TCRγδ , CD19 , HLADR , CD14 , CD45RA , CD39 , IgG , CD141 , CD38 , CD1c | CXCR3 , CD95 |
| 24 | **γδ T-cells** | **Central memory-Naive** | CD3 , TCRγδ , HLADR , CD4 , CD14 , CD39 , IgM , IgG , CD127 , CD141 , CD2 , CD27 , CD28 , CD38 , CD45RA , CD24 | CXCR3 , CD95 |
| 25 | **γδ T-cells** | **Central memory-Naive** | CD3 , TCRγδ , CD4 , CD16 , CD127 , CD2 , CD27 , CD28 | CD95 |
| 26 | **γδ T-cells** | **Naive** | CD3 , CD56 , CD4 , CD16 , CD45RA , CD127 , CD2 , CD27 , CD38 , CD20 | CD95 |
| 27 | **γδ T-cells** | **Naive** | CD3 , CD20 , HLADR , CD8 , CD14 , CD11c , CD45RA , CD39 , IgM , CD127 , CD141 , CD2 , CD24 , CD25 , CD27 , CD28 , CD4 | CXCR3 , CXCR5 , CD95 |
| 28 | **γδ T-cells** | **Naive** | CD3 , TCRγδ , CD20 , CD56 , HLADR , CD4 , CD8 , CD14 , CD16 , CCR7 , CD45RA , CD39 , IgM , IgG , CD127 , CD141 , CD2 , CD24 , CD25 , CD27 , CD28 , CD38 | CXCR3 , CXCR5 , CD95 |
| 29 | **γδ T-cells** | **Naive** | CD3 , TCRγδ , CD20 , CD19 , CD56 , HLADR , CD4 , CD14 , CD16 , CD11c , CCR7 , CD45RA , CD39 , IgM , IgG , CD127 , CD141 , CD2 , CD24 , CD25 , CD27 , CD28 , CD38 , CD123 | CXCR3 , CXCR5 , CD95 |
| 30 | **γδ T-cells** | **Central Memory-Effector Memory** | CD3 , TCRγδ , CD19 , HLADR , CD14 , CD39 , IgG , CD141 , CD38 , CD1c | CXCR3 , CD95 |
| 31 | **CD4+ CD8+ T-cells** |  | CD3 , CD4 , CD8 , CD16 , CD45RA , CD127 , CD2 , CD27 , CD20 | CD95 |
| 32 | **CD4+ CD8+ T-cells** |  | CD3 , CD4 , CD8 , CD16 , CD127 , CD2 , CD27 , CD28 , CD20 | CD95 |
| 33 | **CD4- CD8- T-cells** |  | CD3 , CD2 , CD16 |  |
| 34 | **CD4- CD8- T-cells** |  | CD3 , CD45RA | CD95 |
| 35 | **CD4- CD8- T-cells** |  | CD3 , CD14 , CD141 , CD2 , CD24 , IgM |  |
| 36 | **CD4- CD8- T-cells** |  | CD3 , HLADR , CD45RA , CD27 , CD28 , CD38 | CD95 |
| 37 | **CD4- CD8- T-cells** |  | CD3 , CD20 , HLADR , CD14 , CD16 , CD39 , IgG , CD127 , CD141 , CD24 , CD38 , CD56 | CD95 |
| 38 | **CD4- CD8- T-cells** |  | CD3 , CD20 , CD19 , HLADR , CD14 , CD45RA , CD39 , IgD , IgM ,IgA, CD127 , CD24 , CD27 , CD38 | CXCR5 , CD95 |
| 39 | **CD4- CD8- T-cells** |  | CD3 , CD20 , CD19 , HLADR , CD45RA , CD39 , IgD , IgM , IgA, CD127 , CD24 , CD1c | CXCR5 |
| 40 | **CD4- CD8- T-cells** |  | CD3 , HLADR , CD14 , CD39 , IgG , CD141 , CD38 | CXCR3 , CD95 |
| 41 | **CD4- CD8- T-cells** |  | CD3 , HLADR , CD14 , CD39 , IgG , CD127 , CD141 , CD38 | CD95 |
| 42 | **NKT** | **CD8-** | CD3 , HLADR , CD123 , CD14 , CD16 , CCR7 , CD39 , IgG , CD127 , CD141 , CD2 , CD27 , CD38 , CD56 | CXCR3 , CD95 |
| 43 | **NKT** | **CD8-** | CD3 , CD56 , CD16 , CD127 , CD38 , CD14 , | CD95 |
| 44 | **NKT** | **CD8-** | CD3 , HLADR , CD14 , CD45RA , CD39 , IgG , CD127 , CD141 , CD38 , CD56 | CXCR3 , CD95 |
| 45 | **NKT** | **CD8+** | CD3 , HLADR , CD8 , CD14 , CD45RA , CD39 , IgG , CD127 , CD141 , CD2 , CD27 , CD38 , IgM , CD24 | CXCR3 , CD95 |
| 46 | **NK cells** | **Early** | CD19 , HLADR , CD123 , CD14 , CCR7 , CD39 , IgG , CD127 , CD2 , CD27 , CD38 , CD56 | CXCR3 , CD95 , CXCR5 |
| 47 | **NK cells** | **Early** | CD20 , CD16 , CD11c , CD39 , CD24 , IgG | CXCR3 , CD95 |
| 48 | **NK cells** | **Early-Mature** | CD56 , CD45RA , CD2 , CD16 , CD38 |  |
| 49 | **NK cells** | **Early-Mature** | CD56 , CD16 , CD45RA , CD38 |  |
| 50 | **NK cells** | **Early-Mature** | CD56 , CD45RA , CD127 , CD38 |  |
| 51 | **NK cells** | **Terminal** | CD56 , CD45RA , CD38 , CD16 |  |
| 52 | **NK cells** | **Terminal** | CD56 , CD45RA , CD2 , CD38 |  |
| 53 | **B-cells** | **Naive** | CD20 , CD19 , HLADR , CD45RA , CD39 , IgD , IgM | CXCR5 |
| 54 | **B-cells** | **Naive** | CD20 , CD19 , HLADR , CD45RA , CD39 , IgD , IgM , CD4 | CXCR5 |
| 55 | **B-cells** | **Naive-IgM** | CD20 , CD19 , HLADR , CD45RA , CD39 , IgD , IgM , CD24 , CD1c | CXCR5 |
| 56 | **B-cells** | **IgD- IgM- IgA- IgG-** | CD20 , CD19 , HLADR , CD45RA , CD39 | CXCR5 , CD95 |
| 57 | **B-cells** | **IgD- IgM- IgG+** | TCRγδ , CD19 , HLADR , CD14 , CD39 , IgG , CD141 , CD1c | CXCR3 , CD95 |
| 58 | **Monocytes** | **Mix** | HLADR , CD45RA , CD141 , CD4 , IgG , CD127 | CD95 |
| 59 | **Macrophages** |  | HLADR , CD14 , CD39 , CD141 , IgG , CD1c | CD95 |
| 60 | **pDC** |  | CD3 , CD19 , CD56 , HLADR , CD123 , CD4 , CCR7 , CD45RA , CD39 , IgM , CD127 , CD24 , CD25 , CD27 , CD28 , CD38 | CXCR3 |
| 61 | **cDC** |  | CD20 , HLADR , CD39 , CD141 , CD38 , CD8 |  |
| 62 | **cDC** |  | CD3 , CD19 , HLADR , CD45RA , CD39 , CD27 , CD38 | CD95 |

**SUPPLEMENTARY FIGURE LEGENDS**

**Supplementary Figure 1: Classical or hierarchical gating strategy for total lamina propria and peripheral blood mononuclear cells.**

**A)** The gating strategy is shown to identify the main cellular subsets within the human mucosa. Arrows indicate the relationships across plots, and numbers above each square refer to the immune cell they described. Within singlets viable CD45⁺, (1) basophils and plasmacytoid dendritic cells (pDC) were defined as CD123⁺HLA-DR⁻ and CD123⁺HLA-DR⁺, respectively. Within singlets viable CD45⁺CD123⁻ cells, classical T-cell (CD3⁺ TCRγδ⁻) Tγδ cells (CD3⁺TCRγδ⁺) and no T-cells (CD3⁻TCRγδ⁻) were identified. (2) total Tγδ cells were further characterised based on CD45RA and CCR7 to divide them into Naive (CD45RA⁺CCR7⁺), TEMRA (CD45RA⁺CCR7⁻), Effector Memory (CD45RA⁻CCR7⁻) and Central memory (CD45RA⁻CCR7⁺)(2). The CD3⁺TCRγδ⁻ fraction was further divided into Natural killer T-cells (NKT) (CD3⁺CD56⁺) and classical T-cells (CD3⁺CD56⁻). (3) NKT cells were further characterised based on the expression of CD8. (4) Classical T-cells were divided into CD4⁺, CD8⁺, CD4⁺CD8⁺, and CD4⁻CD8⁻. Regulatory T-cells (Treg) were defined within the CD4⁺ fraction according to the expression of CD127 and CD25 (CD127⁻CD25⁺). In a similar manner, both classical CD4⁺ and CD8⁺ T-cells were further divided into Naive, TEMRA, effector memory and Central memory population based on the expression of CD45RA and CCR7. (5) CD19⁺ and/or CD20⁺ cells (B-cells) were gated out from the CD3⁻TCRγδ⁻ fraction. CD19⁺ CD20⁺/⁻ cells were further characterised as Naïve (IgD⁺IgM⁺), IgD⁻IgM⁺, IgD⁺IgM⁻ and IgD⁻IgM⁻. Within the latter, IgA⁺ and IgG⁺ were further defined. (6) Innate lymphoid cells (ILC) cells were defined as CD3⁻TCRγδ⁻CD19⁻CD14⁻CD123⁻CD2⁺CD127⁺. (7) Natural killer cells (NK) were defined as CD3⁻TCRγδ⁻CD19⁻HLA-DR⁻ cells that express CD16 and/or CD56, and further divided into early NK (CD56⁺CD16⁻), mature NK (CD56⁺ CD16⁺) and terminal NK (CD56⁻CD16⁻). (8) Finally, within CD3⁻TCRγδ⁻CD19⁻ fraction, antigen presenting cells were identified as HLA-DR⁺. Within them, macrophages were identified as CD11c. While the CD11c⁺ fraction was further divided into conventional dendritic cells (cDC) and monocytes based on the expression of CD14 and CD16. Panel **B)** displays the gating approach in the blood. As above, arrows indicate the relationships across plots, and the numbers above each square refer to the described immune cell population. Within singlets viable CD45⁺, (1) basophils and plasmacytoid dendritic cells (pDC) were defined as CD123⁺HLA-DR⁻ and CD123⁺HLA-DR⁺, respectively. Within singlets, viable CD45⁺CD123⁻ cells, T-cells (CD3⁺ TCRγδ⁻), Tγδ cells (CD3⁺TCRγδ⁺) and no T-cells (CD3⁻TCRγδ⁻) were identified. (2) Total Tγδ cells were further characterised based on the expression of CD45RA and CCR7 to divide them into Naive (CD45RA⁺CCR7⁺), TEMRA (CD45RA⁺CCR7⁻), Effector Memory (CD45RA⁻CCR7⁻) and Central memory (CD45RA⁻CCR7⁺). The CD3⁺TCRγδ⁻ fraction was further divided into Natural killer T-cell (NKT) (CD3⁺CD56⁺) and classical T-cells (CD3⁺CD56⁻). (3) NKT cells were further characterised based on the expression of CD8. (4) classical T-cells were divided into CD4⁺, CD8⁺, CD4⁺CD8⁺ and CD4⁻CD8⁻. Regulatory T-cells (Treg) were identified within the CD4⁺ fraction according to the expression of CD127 and CD25 (CD127⁻CD25⁺). Similarly, both classical CD4⁺ and CD8⁺ T-cells were further divided into Naive, TEMRA, effector memory and Central memory populations based on the expression of CD45RA and CCR7. (5) CD19⁺ and/or CD20⁺ cells (B-cells) were gated out from the CD3⁻TCRγδ⁻ fraction. CD19⁺ CD20⁺/⁻ cells were further characterised as Naïve (IgD⁺IgM⁺), IgD⁻IgM⁺ and IgD⁻IgM⁻. Within the latter, IgA⁺ and IgG⁺ were further defined. (6) Innate lymphoid (ILC) cells were defined as CD3⁻TCRγδ⁻CD19⁻CD14⁻CD123⁻CD2⁺CD127⁺. (7) Natural killer cells (NK) were defined as CD3⁻TCRγδ⁻CD19⁻HLA-DR⁻ cells that expressed CD16 and/or CD56 and were further divided into early NK (CD56⁺CD16⁻), mature NK (CD56⁺ CD16⁺) and terminal NK (CD56⁻CD16⁻). (8) Finally, within the CD3⁻TCRγδ⁻CD19⁻HLA-DR⁺ fraction, conventional dendritic cells (cDC) (CD14⁻CD16⁻) and monocytes were gated as non CD14⁻ CD16⁻, with the latter being further divided into classical (CD14⁺CD16⁻), intermediated (CD14⁺CD16⁺) and non-classical (CD14⁻CD16⁺).

**Supplementary Figure 2. Sex-based comparison of mucosal immune profiles**

Unsupervised clustering was conducted using FlowSOM, and relative cluster abundances were compared between sexes, from controls and patients with either Crohn´s disease (CD) or ulcerative colitis (UC), both in lamina propria mononuclear cells (LPMCs) and peripheral blood mononuclear cells (PBMCs). Volcano plots depict differential cluster abundance between males and females for each. Each point represents one FlowSOM-defined cluster; the x-axis indicates log₂ fold change between groups, and the y-axis shows statistical significance (–log₁₀ FDR). Clusters reaching statistical significance are highlighted in green, while their spatial distribution is overlaid onto the original FlowSOM map, together with bar plots showing quantitative differences between males and females. A) Volcano plot showing no differential cluster abundance within lamina propria mononuclear cells (LPMCs) between female and male controls. B) Volcano plot showing differential cluster abundance within LPMCs between ileal CD. C) Clusters significantly enriched in male patients are projected onto the global UMAP representation, allowing visualization of their spatial distribution within the overall cellular landscape. Bar plots display the relative abundance of these clusters in each sex. D) Volcano plot showing no differential cluster abundance within LPMCs between colonic CD. E) The same Volcano approach was also applied within peripheral blood mononuclear cells (PBMCs) between female and male controls. F) Volcano plot showing no differential cluster abundance within PBMCs between ileal CD. G) Volcano plot showing differential cluster abundance within PBMCs between UC patients. H) Clusters significantly enriched in female patients are projected onto the global UMAP representation, allowing visualization of their spatial distribution within the overall cellular landscape. Bar plots display the relative abundance of these clusters in each sex. Differential abundance analysis was performed using edgeR, applying a generalised linear model with multiple-testing correction by the Benjamini–Hochberg FDR method in panels A), B), D), E), F), G) and H). An unpaired two-tailed t-test was applied in panels C) and H). P-value < 0.05 was considered as statistically significant (*p < 0.05; **p < 0.01; ***p < 0.001; ****p < 0.0001).

**Supplementary Figure 3. Age-based comparison of mucosal immune profiles**

Unsupervised clustering was conducted using FlowSOM, and relative cluster abundances were compared between younger and older individuals, from controls and patients with either Crohn´s disease (CD) or ulcerative colitis (UC), in both in lamina propria mononuclear cells (LPMCs) and peripheral blood mononuclear cells (PBMCs). Volcano plots depict differential cluster abundance between males and females for each. Each point represents one FlowSOM-defined cluster; the x-axis indicates log₂ fold change between groups, and the y-axis shows statistical significance (–log₁₀ FDR). Clusters reaching statistical significance are highlighted in green, while their spatial distribution is overlaid onto the original FlowSOM map, together with bar plots showing quantitative differences between males and females. A) Volcano plot showing no differential cluster abundance within lamina propria mononuclear cells (LPMCs) between controls aged >50 years and ≤50 years. B) Volcano plot showing differential cluster abundance within LPMCs between ileal CD patients. C) Clusters significantly enriched in patients aged >50 years are projected onto the global UMAP representation, allowing visualization of their spatial distribution within the overall cellular landscape. Bar plots display the relative abundance of these clusters across age groups. D) Volcano plot showing no differential cluster abundance within LPMCs between colonic CD patients. E) Volcano plot showing no differential cluster abundance within PBMCs between controls. Volcano plot showing no differential cluster abundance within PBMCs between ileal CD (F) and colonic CD (G). Differential abundance analysis was performed using edgeR, applying a generalised linear model with multiple-testing correction by the Benjamini–Hochberg FDR method in panels A), B), D), E), F) and G). An unpaired two-tailed t-test was applied in panel C). P-value < 0.05 was considered as statistically significant (*p < 0.05; **p < 0.01; ***p < 0.001; ****p < 0.0001).

**Supplementary Figure 4. Disease severity–based comparison of mucosal and circulating immune profiles.**

Unsupervised clustering was conducted using FlowSOM, and relative cluster abundances were compared according to endoscopic disease severity in patients with Crohn´s disease (CD) and ulcerative colitis (UC) in both in lamina propria mononuclear cells (LPMCs) and peripheral blood mononuclear cells (PBMCs). Volcano plots depict differential cluster abundance between mild and moderate/severe endoscopic activity for each. Each point represents one FlowSOM-defined cluster; the x-axis indicates log₂ fold change between groups, and the y-axis shows statistical significance (–log₁₀ FDR). Clusters reaching statistical significance are highlighted in green, while their spatial distribution is overlaid onto the original FlowSOM map, together with bar plots showing quantitative differences between disease severity.

A) Volcano plot showing differential cluster abundance, within lamina propria mononuclear cells (LPMCs) between patients with mild and moderate-severe ileal CD. Each point represents one FlowSOM-defined cluster; the x-axis indicates log₂ fold change between severity groups, and the y-axis shows statistical significance (–log₁₀ FDR). Clusters reaching statistical significance after multiple-testing correction are highlighted. B) Clusters significantly enriched in patients with mild ileal CD are projected onto the global UMAP representation, allowing visualization of their spatial distribution within the overall cellular landscape. Bar plots display the relative abundance of these clusters across severity groups. C) The Volcano approach was performed within peripheral blood mononuclear cells (PBMCs) for colonic CD patients showing no differential cluster abundance between severity groups. D) Volcano plot showing differential cluster abundance, within PBMCs between patients with moderate and severe UC. Cluster significantly enriched in patients with moderate UC are projected onto the global UMAP representation (E), allowing visualization of their spatial distribution within the overall cellular landscape. Bar plots display the relative abundance of these clusters across severity groups. Differential abundance analysis was performed using edgeR, applying a generalised linear model with multiple-testing correction by the Benjamini–Hochberg FDR method in panel A), C) and D). An unpaired two-tailed t-test was applied in panels B) and E). P-value < 0.05 was considered as statistically significant (*p < 0.05; **p < 0.01; ***p < 0.001; ****p < 0.0001).
